# Stable nuclear transformation of *Gonium pectorale*

**DOI:** 10.1186/1472-6750-9-64

**Published:** 2009-07-10

**Authors:** Kai Lerche, Armin Hallmann

**Affiliations:** 1 Department of Cellular and Developmental Biology of Plants, University of Bielefeld, Universitätsstr. 25, D-33615 Bielefeld, Germany

## Abstract

**Background:**

Green algae of the family Volvocaceae are a model lineage for studying the molecular evolution of multicellularity and cellular differentiation. The volvocine alga *Gonium *is intermediate in organizational complexity between its unicellular relative, *Chlamydomonas*, and its multicellular relatives with differentiated cell types, such as *Volvox*. *Gonium pectorale *consists of ~16 biflagellate cells arranged in a flat plate. The detailed molecular analysis of any species necessitates its accessibility to genetic manipulation, but, in volvocine algae, transformation procedures have so far only been established for *Chlamydomonas reinhardtii *and *Volvox carteri*.

**Results:**

Stable nuclear transformation of *G. pectorale *was achieved using a heterologous dominant antibiotic resistance gene, the aminoglycoside 3'-phosphotransferase VIII gene (*aphVIII*) of *Streptomyces rimosus*, as a selectable marker. Heterologous 3'- and 5'-untranslated flanking sequences, including promoters, were from *Chlamydomonas reinhardtii *or from *Volvox carteri*. After particle gun bombardment of wild type *Gonium *cells with plasmid-coated gold particles, transformants were recovered. The transformants were able to grow in the presence of the antibiotic paromomycin and produced a detectable level of the AphVIII protein. The plasmids integrated into the genome, and stable integration was verified after propagation for over 1400 colony generations. Co-transformants were recovered with a frequency of ~30–50% when cells were co-bombarded with *aphVIII*-based selectable marker plasmids along with unselectable plasmids containing heterologous genes. The transcription of the co-transformed, unselectable genes was confirmed. After heterologous expression of the luciferase gene from the marine copepod *Gaussia princeps*, which was previously engineered to match the codon usage in *C. reinhardtii*, *Gonium *transformants show luciferase activity through light emission in bioluminescence assays.

**Conclusion:**

Flanking sequences that include promoters from *C. reinhardtii *and from *V. carteri *work in *G. pectorale *and allow the functional expression of heterologous genes, such as the selectable marker gene *aphVIII *of *S. rimosus *or the co-transformed, codon-optimized *G. princeps *luciferase gene, which turned out to be a suitable reporter gene in *Gonium*. The availability of a method for transformation of *Gonium *makes genetic engineering of this species possible and allows for detailed studies in molecular evolution using the unicellular *Chlamydomonas*, the 16-celled *Gonium*, and the multicellular *Volvox*.

## Background

**Figure 1:**
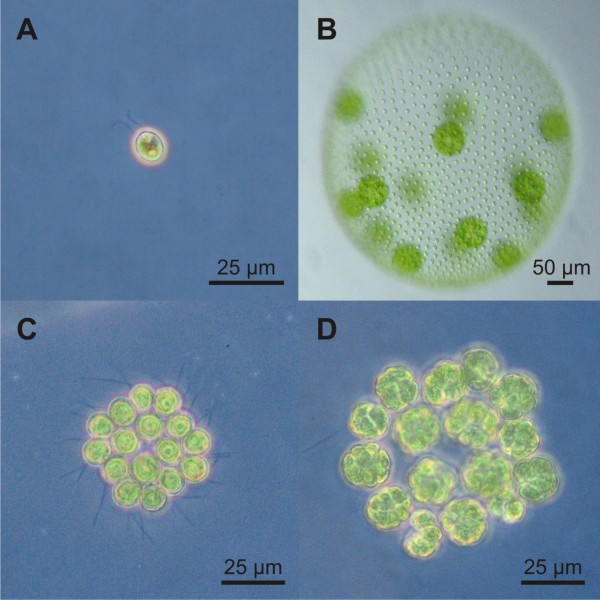
**Wild type phenotypes of three volvocine species**. **(A) ***Chlamydomonas reinhardtii *(strain 137c), a unicellular relative of *Gonium*. **(B) ***Volvox carteri *f. *nagariensis *(female strain Eve), a spheroidal multicellular relative of *Gonium*, which consists of ~2000 small, terminally differentiated, somatic cells at the surface and ~16 reproductive cells in the interior. **(C) **A rhomboidal, flat colony of *Gonium pectorale *(strain SAG 12.85) with 16 cells prior to the onset of cleavage. **(D) **A *G. pectorale *(strain SAG 12.85) colony in the stage of embryogenesis. Each of the 16 cells forms a 16-cell plakea in 4 longitudinal cell cleavages.

In most multicellular lineages, the branch points that lead to multicellularity lie so deep in the past that molecular details of this key step on the path to complex organisms have been obscured by the passage of time. Fortunately, there is an exception. Molecular phylogenetic analysis of volvocine algae showed that the last common ancestor of the unicellular *Chlamydomonas reinhardtii *(Figure 1A) and the multicellular *Volvox carteri*, with its differentiated cell types (Figure 1B), lived only ~200 million years ago [1]. In addition, not only do both unicellular and multicellular forms with differentiated cell types exist within this group, but also forms that are intermediate in organizational complexity between *Chlamydomonas *and *Volvox*, such as *Gonium *(Figure 1C and 1D). A stepwise progression in organismal complexity can be arranged with these and other species of this group, which shows an increase in the number of cells, the degree to which cellular labor is divided between cell types, and the amount of extracellular matrix (for review see [2]). Due to the properties discussed above, volvocine algae attract the interest of researchers who are studying the molecular evolution of multicellularity and cellular differentiation.

The detailed molecular analysis of any species requires its accessibility to genetic manipulation. Among the volvocine algae, transformation procedures have only been established for *C. reinhardtii *[3-5] and *V. carteri *[6]. Similarly, other molecular tools, such as selectable markers and reporter genes, are only available for *C. reinhardtii *and *V. carteri*, and, moreover, only the genomes of these volvocine species have been sequenced (*C. reinhardtii*: [7], *V. carteri*: DOE Joint Genome Institute/JGI, publication in preparation). Unfortunately, no molecular tools and hardly any nucleotide sequence data are available for genera that are intermediate in organizational complexity between *Chlamydomonas *and *Volvox*. Because a molecular analysis of species with intermediate organizational complexity is important for the understanding of molecular evolution, we planned to establish a transformation technique in *Gonium pectorale *to allow for its genetic manipulation. This coenobial (colonial) volvocine green alga builds a slightly convex plate, which typically contains 16 cells in a rather square or rhomboidal arrangement, with four cells in the center and 12 cells in the periphery (Figure 1C) [8,9]. Consistent with its position near the base of the volvocine family tree [10-16], *Gonium *exhibits a number of developmental processes that also occur during the development of its larger and more complex relatives [17] but that are not part of the *Chlamydomonas *developmental program.

An important precondition for genetic transformation is the availability of an appropriate selectable, preferably dominant, marker. Two such genes have been shown to work in both *V. carteri *and *C. reinhardtii*, which are the *ble *gene of *Streptoalloteichus hindustanus*, which mediates resistance to zeocin, and the aminoglycoside 3'-phosphotransferase VIII gene (*aphVIII*) of *Streptomyces rimosus*, which confers resistance to paromomycin [18-21].

Both genes are dominant because they add new, selectable features to transformants, and therefore do not require strains with particular auxotrophic defects as recipients. The Ble protein is a binding protein, which requires a 1:1 ratio of Ble and zeocin. In contrast, the enzyme aminoglycoside 3'-phosphotransferase VIII achieves high-affinity phosphorylation of paromomycin, and, as an enzyme, it works at lower concentrations than a simple binding protein. Therefore, the *aphVIII *gene was determined to be more appropriate for transformation.

The expression of foreign genes is mostly carried out by using endogenous 5'-UTRs, including promoter sequences, and endogenous 3'-UTRs [18-21]. If the genome of a target species has been sequenced, appropriate 5'- and 3'-sequences can be found with bioinformatic tools and can be easily amplified by PCR. Unfortunately, the genome of *Gonium *or other volvocine species, except for *C. reinhardtii *and *V. carteri*, has not been sequenced. Although there is limited sequence information from a few coding sequences for these species, there is no sequence information for the corresponding 5'- and 3'-UTRs, and no information is available as to whether the genes are strongly and constitutively expressed. Because 5'- and 3'-UTRs are weakly conserved even between closely related species, sequence information from related species is not appropriate to allow for amplification of UTRs by standard PCR. In addition, cloning and sequencing of flanking sequences is time consuming. Thus, our strategy was to test flanking sequences that were derived from species closely related to *Gonium*. Previously, it has been shown that flanking sequences from *C. reinhardtii *could work in *V. carteri *and vice versa [22]. We decided to utilize such flanking sequences in *Gonium *transformation experiments with glass beads or with two different particle guns.

In order to allow the application of efficient molecular genetic approaches in *G. pectorale*, appropriate reporter genes that can express functional proteins within this species are also required. The candidate reporter genes that we tested included the arylsulfatase (*ars*) gene from *V. carteri *[23,24], the tagged heat shock protein 70A (*hsp*70A) gene from *V. carteri *[25,26], and the luciferase (*luc*) gene from *G. princeps*, which had been previously codon-optimized for expression in *C. reinhardtii *[27].

Here we report the stable nuclear transformation of *G. pectorale *by particle gun bombardment using the heterologous, dominant antibiotic resistance gene *aphVIII *of *S. rimosus *fused to heterologous 3'- and 5'-untranslated flanking sequences, including promoters, from *C. reinhardtii *and *V. carteri*. We also show that the heterologously expressed luciferase gene from *G. princeps*, which was codon-optimized for *C. reinhardtii*, can serve as a suitable reporter gene in *Gonium*.

## Results

### Phylogenetic analysis of utilized *Gonium pectorale* strains

The identity of the utilized wild type *Gonium pectorale *strains SAG 12.85, CCAP 32/14 and NIES-1710 was verified in a phylogenetic analysis using four DNA sequences (*psa*A, *psa*B, *rbc*L and ITS 1/5.8S rRNA/ITS 2) [10-16]. Both background and procedures of the phylogenetic analysis are described in Additional File 1, alignments are shown in Additional Files 2, 3, 4 and 5, calculations of sequence identities are given in Additional Files 6 and 7, and phylogenetic trees are shown in Additional Files 8, 9, 10, 11 and 12.

### Preliminary transformation studies using *aphVIII*-based selectable marker plasmids

**Figure 2:**
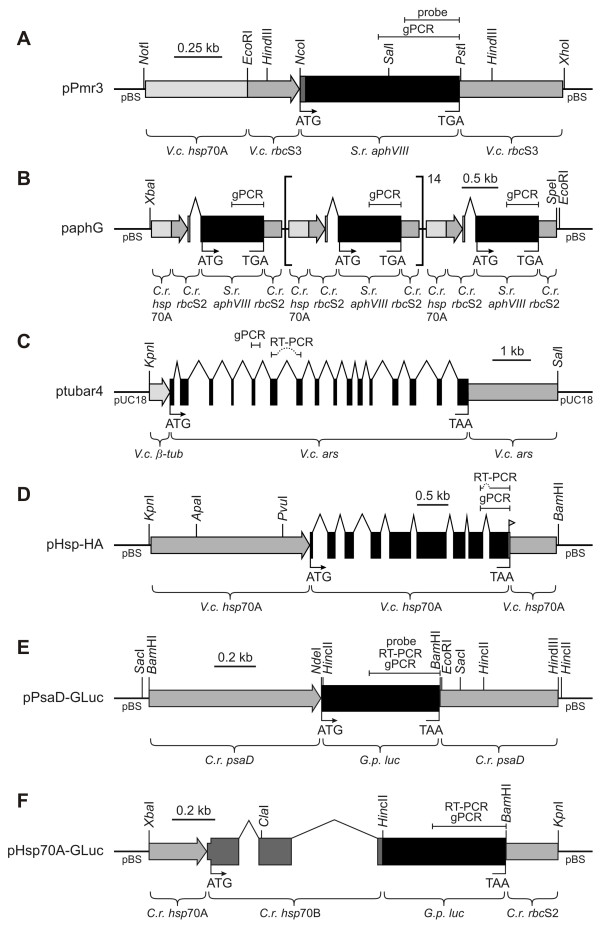
**Schematic diagram of chimeric selectable marker plasmids and non-selectable, co-transformed plasmids**. Plasmids pPmr3 **(A) **and paphG **(B) **are selectable plasmids, and plasmids ptubar4 **(C)**, pHsp-HA **(D)**, pPsaD-GLuc **(E)**, and pHsp70A-GLuc **(F) **are non-selectable, co-transformed plasmids. Introns in **(B-D) **and **(F) **are indicated by right-angled lines. The *hsp*70A gene in **(D) **was tagged with a sequence coding for the HA-epitope (little flag). All constructs are within pBluescript II (pBS) or pUC18 vectors. The positions of DNA fragments that were amplified by genomic PCR (gPCR) or RT-PCR are indicated. Positions of probes that were used in Southern blots are indicated (probe). The plasmids are described in detail in the Methods section. *V.c.*, *Volvox carteri*; *C.r.*, *Chlamydomonas reinhardtii*; *S.r.*, *Streptomyces rimosus*; *G.p.*, *Gaussia princeps*;* ars*, arylsulfatase gene; *β-tub*, β2-tubulin promoter; *luc*, luciferase gene.

For transformation experiments with *G. pectorale*, the aminoglycoside 3'-phosphotransferase VIII gene (*aphVIII*) of *Streptomyces rimosus *was used as a selectable marker. In the plasmid pPmr3, the *aphVIII *gene is under the control of a *V. carteri hsp*70A-*rbc*S3 hybrid promoter, and the 3'-UTR is derived from the *V. carteri rbc*S3 gene [21] (Figure 2A). In plasmid paphG the *aphVIII *gene is under control of a *C. reinhardtii hsp*70A-*rbc*S2 hybrid promoter, and the 3'-UTR is derived from the *C. reinhardtii rbc*S2 gene [22] (Figure 2B). This DNA construct also contains intron 1 of the *rbc*S2 gene 42 bp upstream of the translation start codon. The plasmid paphG includes not just one copy of this hybrid *aphVIII *gene construct but 16 repeated *aphVIII *cassettes, this resulting in a higher gene dosage [22].

We attempted to transform *G. pectorale *(SAG 12.85) by using both plasmids and the glass bead-mediated transformation protocol (untreated or pretreated with proteases to reduce cell wall material) [28] and protocols using a homemade particle gun [6]. After this treatment and a 48-h recovery period, cells were spread on agar plates with paromomycin, but no transformants were obtained. However, we realized in control experiments that we were not even able to grow the wild type cells on agar plates without paromomycin if only a few viable cells were spread per plate. Therefore, in all the following experiments, selection for paromomycin-resistance was performed in liquid medium.

In parallel to the *aphVIII*-based selectable marker plasmids, a promoterless *aphVIII *construct was tested, since a similar promoter-trapping attempt had worked in *Chlamydomonas *[20,29]. Using the particle gun and the promoterless *aphVIII*, a single paromomycin-resistant transformant was obtained (data not shown). This transformant was retained and was stable, and its paromomycin-resistance has persisted over three years. Unfortunately, about ten attempts to reproduce this result have failed.

### Recovery of transformants after biolistic transformation

**Figure 3:**
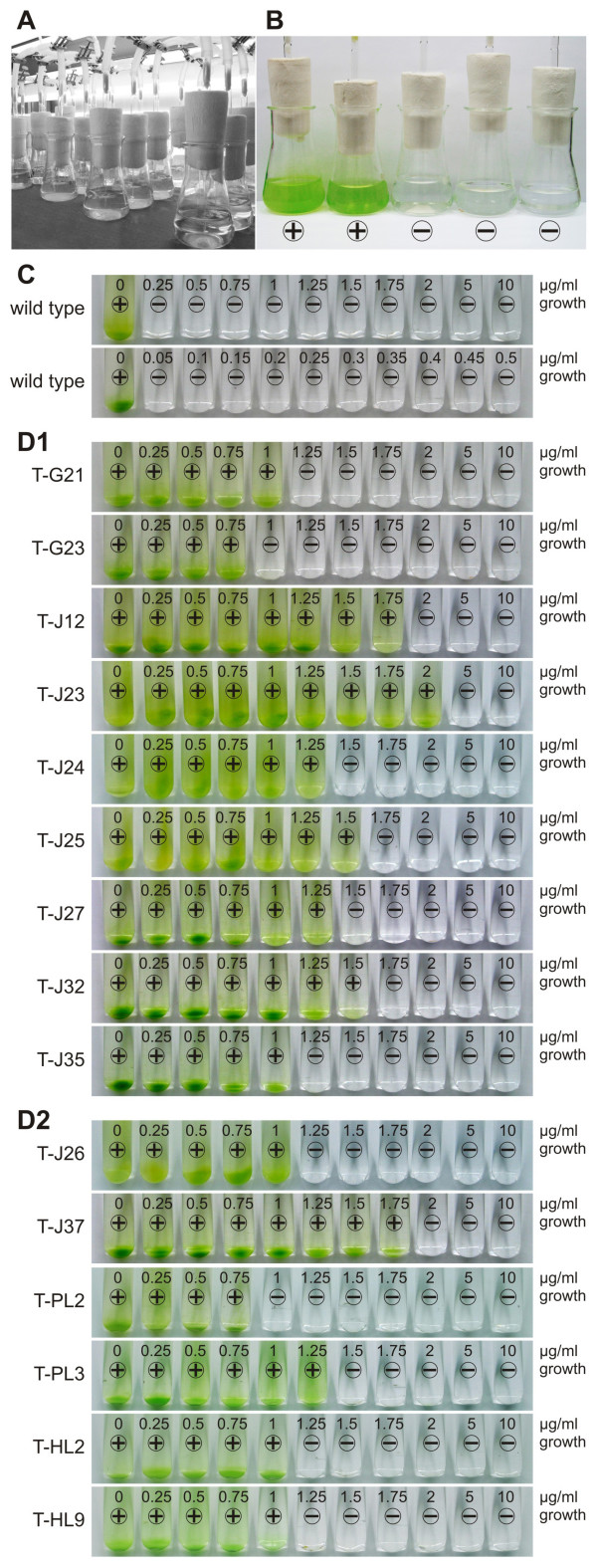
**Analysis of paromomycin resistance in wild type and transgenic *G. pectorale *strains**. **(A) **After particle bombardment, treated organisms were distributed in Erlenmeyer flasks, aerated, and illuminated in a dark/light cycle. Paromomycin (1 μg/ml) was added after 48 h. **(B) **Appearance 12 days after particle bombardment exemplified by five flasks. **(C) **and **(D)**: For detailed analysis of resistance, identical quantities of cells were incubated with increasing concentrations of paromomycin for eight days. **(C) **Analysis of wild type *G. pectorale *(upper row: 0–10 μg; lower row: 0–0.5 μg paromomycin/ml). **(D) **Analysis of transgenic strains (0–10 μg paromomycin/ml). **(D1) **Strains transformed with the selectable marker plasmids paphG (T-G...) or pPmr3 (T-J...). (**D2**) Strains co-transformed with the selectable marker plasmid pPmr3 along with the non-selectable plasmids ptubar4 (T-J...), pPsaD-GLuc (T-PL...), or pHsp70A-GLuc (T-HL...). **(B-D) **plus sign (+), flasks/vials with paromomycin-resistant green organisms; minus sign (-), cleared flasks/vials with some white remains of dead organisms.

After these unsuccessful attempts, a biolistic method for transformation, which was modified from previous protocols [4,6], in combination with the *aphVIII*-based plasmids pPmr3 and paphG and selection in liquid medium, made the reproducible recovery of *G. pectorale *transformants possible. Optimization of the transformation protocol was done by changing several parameters systematically (Table 1). In the most successful protocol, gold microprojectiles (0.6 μm in diameter) were coated with plasmid DNA by a modified ethanol precipitation in the presence of CaCl_2 _and spermidine (see Methods). Logarithmically growing *G. pectorale *cells (strain SAG 12.85) were spread on a cellulose acetate membrane filter, with all liquid removed, and a PDS-1000/He biolistic device (Bio-Rad, Hercules, CA) was used to introduce the plasmid-coated microprojectiles into the cells by high-velocity bombardment. An overview of the most successful combination of transformation parameters is provided in Table 2. After microprojectile bombardment, cells were distributed evenly among 10 flasks (Figure 3A) and incubated for 48 h under standard conditions without selective pressure. After the addition of 1 μg paromomycin/ml, the cultures clarified within 24 h due to the death of most cells. Re-greening of culture flasks within 9–16 days of further incubation indicated the presence of antibiotic-resistant transformants (Figure 3B). Due to the cultivation in liquid medium, we could not easily determine whether the transformed organisms were descendants of a single transformant or of more than one transformant. Thus, we assumed a yield of only one transformant per flask, even if there could be more than one. Based on this assumption, the transformation efficiency was estimated to be ~6.6 × 10^-7 ^and ~1.1 × 10^-7 ^when the plasmids pPmr3 and paphG were used as selectable marker plasmids, respectively. Similar transformation efficiencies were achieved when we repeated the experiments on different wild type strains (NIES-1710 and CCAP 32/14).

**Table 1:** Influence of different parameters on transformation efficiency.

**Parameter and parameter specification**	**Efficiency**
**Transformation method**	
glassbeads (untreated cells)	**-**
glassbeads (pretreated with protease)	**-**
particle gun (homemade, without vacuum chamber)	**+ +**
particle gun (PDS-1000/He biolistic device, with vacuum chamber)	**+ + + + +**

**Material of microprojectiles**	
gold	**+ + + + +**
tungsten	**+ +**

**Size of microprojectiles**	
0.6 μm in diameter	**+ + + + +**
1.0 μm in diameter	**+**
1.6 μm in diameter	**-**

**Selectable marker plasmid**	
paphG	**+ +**
pPmr3	**+ + + + +**
promoterless *aphVIII*	**(+)**

**Coating of microprojectiles**	
plasmid-DNA/microcarrier/NaAc/EtOH-precipitation	**+ + +**
plasmid-DNA/microcarrier/CaCl_2_/spermidine/EtOH-precipitation	**+ + + + +**

**Target cells**	
resuspended in as less liquid as possible; spread in an empty Petri dish	**-**
immobilized on moist filter paper; more or less free of liquid	**+ + +**
immobilized on cellulose acetate membrane filter; almost free of liquid	**+ + + + +**

**Burst pressure of rupture disks**	
450 psi	**-**
650 psi	**-**
900 psi	**+**
1100 psi	**+ + + + +**
1350 psi	**+ +**

**Rupture disk-macrocarrier distance**	
8 mm	**+ + + + +**
16 mm	**+ +**

**Macrocarrier-stopping screen distance**	
7 mm	**+ + + + +**
12 mm	**+ + + +**
18 mm	**+ + +**

**Stopping screen-target cell distance**	
6 cm	**+ + + + +**
11 cm	**+ +**
18 cm	**-**

**Chamber evacuation**	
(almost) no evacuation	**+ +**
15 inch Hg	**+ +**
27 inch Hg	**+ + + + +**

**Cultivation after particle bombardment**	
on agar plates	**-**
in liquid medium	**+ + + + +**

Within each subgroup, the condition that gave the largest number of transformants was set to "+ + + + +"; there is a "-" when no transformants were recovered. The data allow just a qualitative estimation, because a) they were obtained under varying other conditions in the course of optimization, b) poor results disqualified a condition without rechecking, and c) some of the transformants might be false-positives as only a few were analyzed in detail.

**Table 2:** Most successful combination of parameters for *G. pectorale *transformation using a PDS-1000/He biolistic device.

**Parameter**	**Parameter specification**
material of microprojectiles	gold
size of microprojectiles	0.6 μm in diameter
selectable marker plasmid	pPmr3
coating of microprojectiles	plasmid-DNA/microcarrier/CaCl_2_/spermidine/EtOH-precipitation
target cells	immobilized on cellulose acetate membrane filter; almost free of liquid
burst pressure of rupture disks	1100 psi
rupture disk-macrocarrier distance	7 mm
macrocarrier-stopping screen distance	8 mm
stopping screen-target cell distance	6 cm
chamber evacuation	27 inch Hg
cultivation after particle bombardment	in liquid medium

For detailed analyses, the transformants were re-isolated to ensure uniform genetic conditions (see Methods).

### Paromomycin resistance of transformants

As a reference, wild type *G. pectorale *cultures (strain SAG 12.85) were examined for their sensitivity towards paromomycin. A very low concentration of 0.05 μg paromomycin/ml was able to kill all wild type cells (Figure 3C). Transformants were tested at antibiotic concentrations from 0.25 to 10 μg/ml, so that the lowest antibiotic concentration was five times higher than the deadly dose for wild type cells. *Gonium *transformants that were generated using the selectable marker plasmid paphG were able to tolerate 0.75 – 1.0 μg paromomycin/ml (Figure 3D1), which was 15 – 20 times higher than the concentration that kills all wild type cells. Transformants that were produced using pPmr3 tolerated 0.75 – 2.0 μg paromomycin/ml (Figure 3D1), which was 15 – 40 times higher than the concentration that kills all wild type cells. Similar results were obtained when pPmr3 was co-transformed with the non-selectable plasmids described below (Figure 3D2).

### Stable integration of plasmid DNA into the genome of transformants

**Figure 4:**
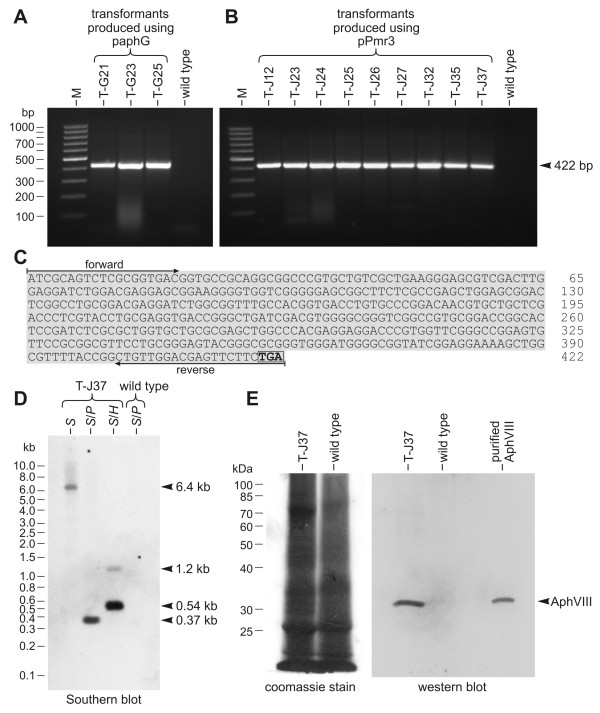
**Detection of the *aphVIII *gene and AphVIII protein in transformants**. **(A-C) **Paromomycin-resistant transformants and the parent wild type strain were analyzed by genomic PCR. Only transformants that were produced using plasmid paphG **(A) **or pPmr3 **(B) **were expected to yield a 422 bp fragment of the *aphVIII *gene. Lane M, molecular weight marker. **(C) **Sequence obtained from cloned *aphVIII *fragments. The positions of the primers and the stop codon (boxed) are indicated. **(D) **Southern blot analysis of genomic DNA from transformant T-J37, which was produced using plasmid pPmr3, and from the parent wild type strain. The blot was probed using an *aphVIII *fragment. *H*, *Hin*dIII; *P*, *Pst*I; *S*, *Sal*I. **(E) **Western blot analysis of cell lysates of T-J37 and the parent wild type strain (middle), and purified AphVIII protein as a reference (right). The anti-AphVIII antibody was used for detection. As a control, the cell lysates were also stained with coomassie blue (left).

To verify the integration of plasmid DNA into the genome, genomic DNA of strains transformed with paphG or pPmr3 was isolated, and the wild type *Gonium *strain SAG 12.85 was used as a control. Using these different genomic DNA isolates as templates, PCR amplification of a fragment of the *aphVIII *gene confirmed transformation. The relative positions of the amplified DNA fragments are indicated in Figure 2A and 2B. Based on the known sequence, the size of the PCR fragment was predicted to be 422 bp. Paromomycin-resistant transformants, which were generated either with paphG (Figure 4A) or pPmr3 (Figure 4B), yielded a PCR fragment of the expected size, whereas the wild type strain gave no such fragment, as expected. Sequence analysis demonstrated that all the amplified fragments were identical to the sequence of the *aphVIII *fragment (Figure 4C).

In addition, genomic DNA from transformants and from the parent wild type strain was analyzed on Southern blots for the presence of the *aphVIII *sequence. Sequences hybridizing to a 282 bp fragment from the coding region of the *aphVIII *gene were detectable in transformants, but not in the parent strain. A Southern blot of genomic DNA from T-J37, which is a transformant that was produced with plasmid pPmr3, and of genomic DNA from the parent wild type strain SAG 12.85 is shown in Figure 4D. The total size of plasmid pPmr3 is 5.1 kb, which includes the pBluescript II vector backbone [21]. Based on the sequence of pPmr3 (see GenBank: AY429514) and the position of the probe (Figure 2A), a signal from a 0.37 kb fragment was expected in the *Sal*I/*Pst*I digest, if pPmr3 integrated into the genome by recombination outside the coding region. Such a fragment was identified in T-J37 DNA, and no such fragment, or any other fragment, was detectable in the parent wild type strain. In T-J37, a 0.54 kb fragment was detected in the *Sal*I/*Hin*dIII digest, as expected from the plasmid sequence, and this shows that the sequences ~0.3 kb upstream and ~0.2 kb downstream of the stop codon were integrated as they appear in the plasmid pPmr3. The *Sal*I/*Hin*dIII-lane also contained a weak 1.2 kb band; this is a known *Hin*dIII – *Hin*dIII fragment (Figure 2A), which arises from incomplete *Sal*I-digestion in the *Sal*I/*Hin*dIII double digest. This 1.2 kb fragment contained the complete *aphVIII *coding region, which included ~0.2 kb upstream of the start codon and ~0.2 kb downstream of the stop codon. In addition to these three genomic fragments that were arranged as predicted from the original plasmid pPmr3, a 6.4 kb *Sal*I-fragment was detected. Plasmid pPmr3 contains only a single *Sal*I-site ~0.4 kb upstream of the stop codon. The second *Sal*I-site of the 6.4 kb fragment must, therefore, be located within the genomic sequence that flanked the integrated plasmid. In addition to the pPmr3-transformants, the *aphVIII *gene was detected on Southern blots with genomic DNA from transformants that were produced with plasmid paphG (data not shown).

In Southern blot results from nine independent transformants, six of them were judged to have a single copy of the plasmid in the genome. The other three seemed to have two or three copies as well as DNA fragments of sizes that were not explainable. Therefore, integration into the genome might have been more complicated in these transformants. The Southern blot results suggest that frequently only one copy of the plasmid integrates into the genome.

### Detection of AphVIII protein in transformants

Based on the amino acid sequence, which was deduced from the cDNA sequence, an AphVIII protein of 30.97 kDa was predicted. In western blot analysis, an anti-AphVIII antibody detected a ~31 kDa band in strains that were transformed with *aphVIII*-based selectable marker plasmids (Figure 4E). As expected, no AphVIII protein was detectable in the parent wild type *Gonium *strain SAG 12.85.

### Stable co-transformation of unselectable genes

**Figure 5:**
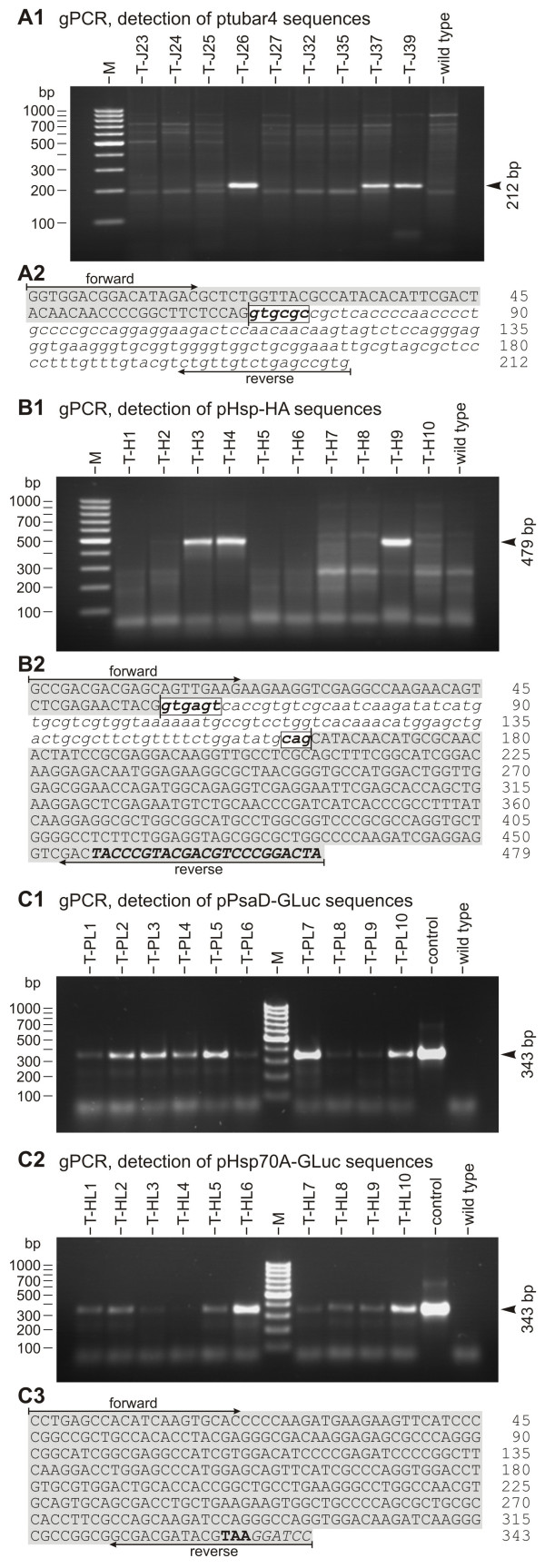
**Demonstration of co-transformation of heterologous genes by genomic PCR**. PCRs were done using genomic template DNA of paromomycin-resistant transformants co-bombarded with the non-selectable plasmids ptubar4 **(A1)**, pHsp-HA **(B1)**, pPsaD-GLuc **(C1)**, or pHsp70A-GLuc **(C2)**. Primers were specific for the co-bombarded genes. Co-transformants were expected to yield gene fragments of the *V. carteri *arylsulfatase (*ars*) (212 bp, **A1**), the *V. carteri hsp*70A (479 bp, **B1**), or the *Gaussia *luciferase (*luc*) (343 bp, **C1 **and **C2**). Sequences obtained from cloned fragments are shown in **A2 **(*ars*, ptubar4 co-transformants), **B2 **(*hsp*70A, pHsp-HA co-transformants), and **C3 **(*luc*, pPsaD-GLuc and pHsp70A-GLuc co-transformants). **(A1, B1, C1, C2) **The parent wild type strain was analyzed as a control; lane M, molecular weight marker. **(C1, C2) **Control, the co-transformed plasmid was used as a template. **(A2, B2, C3) **Primer positions are indicated; upper case (grey background), exon; lower case and italics (white background), intron; boxed: intron ends. **(B2) **Upper case, italics, and bold: HA-tag. **(C3) **Bold, stop codon; italics, artificial *Bam*HI site.

The usefulness of a transformation system also depends on the convenience with which co-transformations with unselectable genes can be achieved, as most genes of interest do not allow for direct selection. Construction of transformation vectors is more laborious and time-consuming if both the selectable and the unselectable gene must be inserted into a single plasmid, particularly when the genes are quite large. Therefore, co-transformation using two separate plasmids is preferable, if possible. To test this, wild type *G. pectorale *organisms (strain SAG 12.85) were co-bombarded using plasmid pPmr3 (Figure 2A), which contains the *aphVIII *gene for selection, and the following four unselectable plasmids: 1) plasmid ptubar4 (Figure 2C), in which the arylsulfatase (*ars*) gene from *V. carteri *is under control of the β2-tubulin promoter from *V. carteri *[23,24,30]; 2) plasmid pHsp-HA (Figure 2D), in which the tagged *V. carteri *heat shock protein 70A (*hsp*70A) gene is under control of its own promoter [25]; 3) plasmid pPsaD-GLuc (Figure 2E), in which the luciferase (*luc*) gene from *G. princeps *is under control of the *psaD *promoter from *C. reinhardtii *and in which the *luc *gene was codon-optimized for expression in *C. reinhardtii *[27]; and 4) plasmid pHsp70A-GLuc (Figure 2F), in which the luciferase (*luc*) gene from *G. princeps*, which is again codon-optimized for *C. reinhardtii*, is fused to the first three exons of the *hsp*70B gene of *C. reinhardtii *and in which this hybrid gene is under control of the *hsp*70A promoter from *C. reinhardtii *[27]. Paromomycin-resistant transformants were obtained from all the co-bombardments, and, in all transformants, the presence of the *aphVIII *gene was verified by genomic PCR. The co-bombarded plasmid was detectable in ~30–50% of the paromomycin-resistant transformants regardless of which co-bombarded plasmid was used. The results of representative PCRs using genomic template DNA of different paromomycin-resistant transformants co-bombarded with the selectable plasmid pPmr3 and the non-selectable plasmids ptubar4, pHsp-HA, pPsaD-GLuc, or pHsp70A-GLuc are shown in Figure 5. The primers are specific for each of the co-bombarded genes. The relative positions of the amplified DNA fragments are indicated in Figure 2C, D, E, and 2F, respectively. In each co-transformation experiment, fragments of the expected sizes of 212 bp (Figure 5A1), 479 bp (Figure 5B1), 343 bp (Figure 5C1), and 343 bp (Figure 5C2) were amplified from some of the paromomycin-resistant transformants (Figure 5). The sequence of the amplified fragments with the correct size was confirmed by sequence analysis in each of the four co-transformation experiments, and all amplified fragments showed the correct sequence (Figure 5A2, B2, and 5C3).

### Transcription of co-transformed, unselectable genes

The RT-PCR technique was used to verify the existence of heterologous transcripts in *Gonium *transformants. Oligonucleotide primers were selected, and the following cDNA-fragments were predicted: a 265 bp *ars *fragment from ptubar4 co-transformants, a 374 bp *hsp*70A fragment from pHsp-HA co-transformants, and a 343 bp *luc *fragment from both pPsaD-GLuc and pHsp70A-GLuc co-transformants. The relative positions of these cDNA fragments are indicated in Figure 2C, D, E, and 2F, respectively.

**Figure 6:**
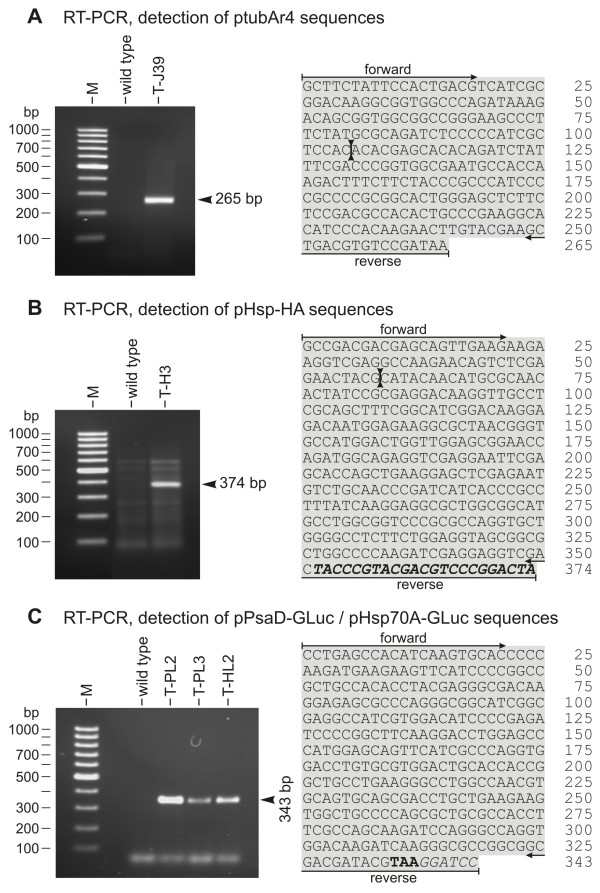
**Demonstration of transcription of co-transformed, heterologous genes by RT-PCR**. RNA from paromomycin-resistant transformants that were co-transformed with the non-selectable plasmids ptubar4 **(A**, T-J39**)**, pHsp-HA **(B**, T-H3**)**, pPsaD-GLuc **(C**, T-PL2, T-PL3**)**, or pHsp70A-GLuc **(C**, T-HL2**) **was reverse transcribed and products were amplified by PCR using primers specific for the heterologous genes. Co-transformants were expected to yield cDNA fragments of the *V. carteri *arylsulfatase (*ars*) (265 bp, **A**), the *V. carteri hsp*70A (374 bp, **B**), or the *Gaussia *luciferase (*luc*) (343 bp, **C**). Sequences obtained from cloned fragments are shown in **A **(*ars*, co-transformant T-J39), **B **(*hsp*70A, co-transformant T-H3), and **C **(*luc*, co-transformants T-PL2, T-PL3, and T-HL2). **(A-C) **The parent wild type strain was analyzed as a control; lane M, molecular weight marker. The positions of the primers and the former positions of introns (two connected arrowheads) are indicated in the sequences. **(B) **Italics and bold, HA-tag sequence. **(C) **Bold, stop codon; italics, artificial *Bam*HI site.

All RT-PCRs with the co-transformants yielded cDNA fragments with the expected sizes, and DNA sequence analysis demonstrated the correctness of the sequences (Figure 6). If introns were present at the corresponding position of a gene, the intron sequences were spliced correctly.

The RT-PCR experiments demonstrated that the promoters of the *Volvox *β2-tubulin and *hsp*70A genes and of the *Chlamydomonas psaD *and *hsp*70A genes mediate transcription of co-transformed, heterologous genes in *Gonium*.

### Analysis of heterologous protein expression in co-transformants

Transformants that were generated through co-transformation with the *ars *plasmid ptubar4 were analyzed for arylsulfatase activity. The transformants were grown in sulfur-sufficient medium because endogenous *ars *genes are induced by sulfur deficiency. Under these conditions, we were not able to detect any arylsulfatase activity in transformants (data not shown). Likewise, we were not able to detect the HA-tagged Hsp70A protein in transformants that were co-transformed with plasmid pHsp-HA, since there was no detectable signal in western blots using anti-HA antibodies (data not shown).

**Figure 7:**
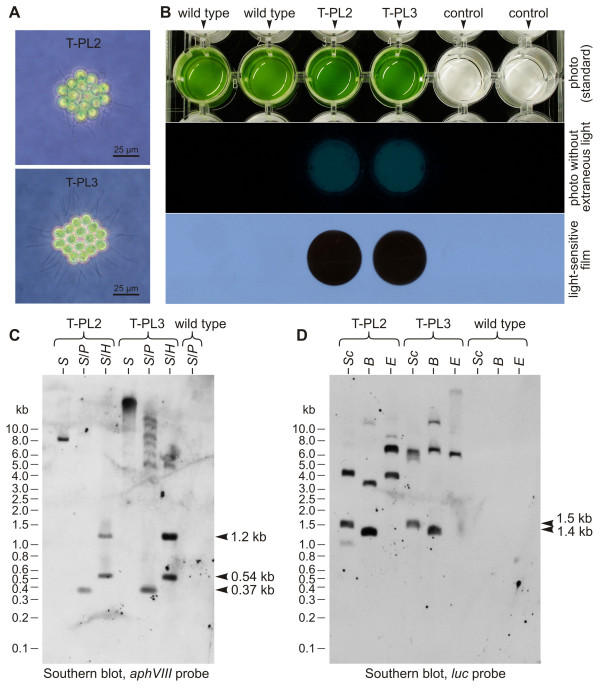
**Transformants expressing the codon-optimized luciferase gene from *G. princeps*: phenotype, detection of luciferase activity, and Southern blot analysis**. **(A) **Phenotypes of transformants T-PL2 and T-PL3, which were co-transformed with the selectable marker plasmid pPmr3 and the non-selectable plasmid pPsaD-GLuc. **(B) **Luciferase assay of wild type and transformants T-PL2 and T-PL3 (at 20°C). Control: medium only. Upper row: standard photo showing the assay setup. Middle row: photo without any extraneous light after addition of the coelenterazine substrate. Lower row: exposure to a light-sensitive film for 30 s. **(C) **and **(D)**: Southern blot analysis of genomic DNA from transformants T-PL2 and T-PL3 and from the parent wild type strain. The blot in **(C) **was probed using a fragment of the *aphVIII *gene, the blot in **(D) **was probed using a fragment of the luciferase (*luc*) gene. *B*, *Bam*HI; *E*, *Eco*RI; *H*, *Hin*dIII; *P*, *Pst*I; *S*, *Sal*I; *Sc*, *Sac*I.

Transformants that were generated through co-transformation with plasmid pPsaD-GLuc were analyzed for luciferase activity. In the light microscope, all transformants show a wild type phenotype (Figure 7A) without any detectable morphological differences in comparison to the parent strain SAG 12.85 (Figure 1C), but when the coelenterazine substrate is added to the lysates of these transformants, a glow is visible to the naked eye in the darkroom (Figure 7B). Exposure to a light-sensitive film allows a simple qualitative analysis of chemiluminescence (Figure 7B).

Genomic DNA from these transformants was analyzed on Southern blots to estimate the number of copies that integrated into the genome. Using the *aphVIII *probe (Figure 2A), we looked for fragments that originate from integration of plasmid pPmr3 into the genome, and we obtained fragments of the expected sizes (Figure 2A, 0.37 kb, 0.54 kb, and 1.2 kb) in transformants, but not in the parent wild-type strain (Figure 7C), as described above. The co-transformed plasmid pPsaD-GLuc, which contains the luciferase gene, has a total size of 5.0 kb that includes the pBluescript II vector backbone [27]. The *luc *probe (Figure 2E) also detected fragments of the expected sizes (Figure 2E, 1.4 kb *Sac*I fragment, 1.5 kb *Bam*HI fragment) in transformants, whereas no fragments were detectable in the parent wild-type strain (Figure 7D). We were not able to assign the other fragment sizes observed in transformants due to the unknown flanking sequences. However, the relatively low number of bands in each restriction digest of genomic DNA from the two transformants that are shown in Figure 7D and from four other transformants that were analyzed, suggests that there are only ~1–3 integrations of the co-transformed plasmid, so a high gene dosage through a large number of integrations can be excluded.

Transformants that were generated through co-transformation with plasmid pHsp70A-GLuc were also analyzed qualitatively for luciferase activity, and several of these transformants showed chemiluminescence (data not shown).

**Figure 8:**
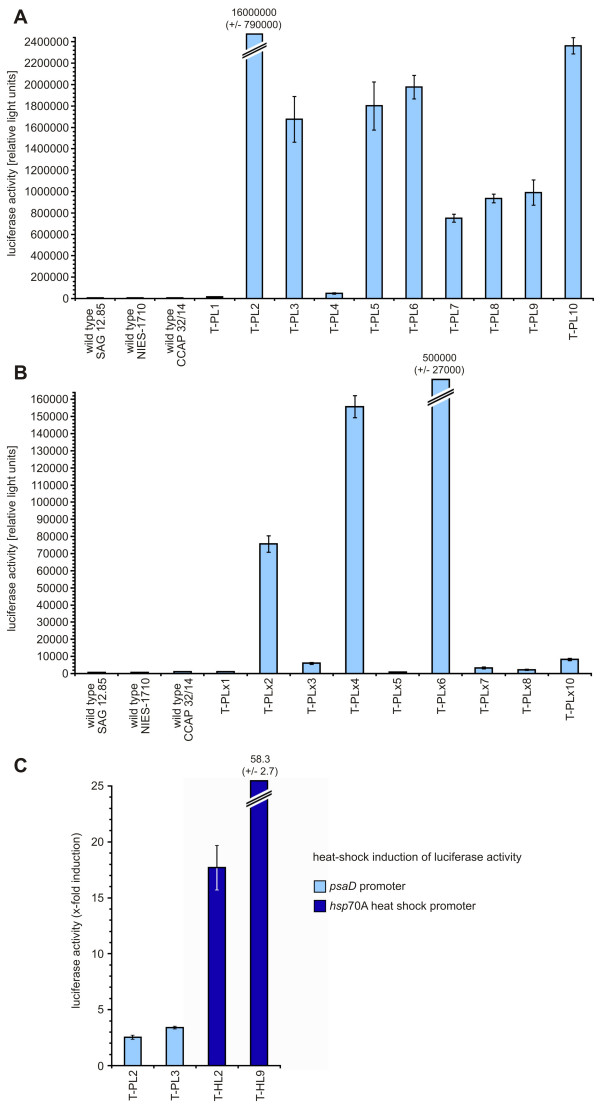
**Quantitation of luciferase activity in transformants expressing the luciferase gene**. Luciferase activity was assayed using a luminescence counter. The bars represent the mean of three independent experiments. The standard deviation is indicated. **(A) **Luciferase activity of pPsaD-GLuc-derived transformants (T-PL...) is compared to wild type strains; T-PL-transformants descend from the wild type strain SAG 12.85. **(B) **Luciferase activity of pPsaD-GLuc-derived transformants (T-PLx...) is compared to wild type strains; T-PLx-transformants descend from a different wild type strain, CCAP 32/14. **(C) **Induction of luciferase activity in heat-shocked versus non-heat-shocked transformants. In pPsaD-GLuc-derived transformants (T-PL...) the luciferase gene is driven by the *psaD *promoter. In pHsp70A-GLuc-derived transformants (T-HL...) the luciferase gene is under control of the *hsp*70A heat shock promoter. Transformants were subject to a temperature shift from 23 to 36°C for 1 h. After a 1 h recovery phase at 23°C, cells were lysed and luciferase activity was assayed. As a reference, non-heat-shocked transformants were analyzed in the same way.

To obtain quantitative expression data of the co-transformed *luc *gene, we analyzed the bioluminescence of pPsaD-GLuc-derived transformants using a luminometer (Figure 8A); in pPsaD-GLuc, the *luc *gene is under control of the *psaD *promoter from *C. reinhardtii*. These transformants descend from the *G. pectorale *wild type strain SAG 12.85. For comparison, we also generated pPsaD-GLuc-derived transformants using a different parent *G. pectorale *wild type strain, CCAP 32/14. The amount of light emission was extremely different in the transformants from both parent strains. Several re-examinations showed that these differences are permanent (Figure 8A and 8B). It is known from many other species that expression of transgenes is strongly influenced by the unpredictable effects of elements at the site of chromosomal integration [31]. For unknown reasons, most of the SAG 12.85-derived transformants showed significantly higher luciferase expression rates than the CCAP 32/14-derived transformants (Figure 8A and 8B).

Because the promoter of *hsp*70A is heat-shock inducible in *C. reinhardtii*, we wanted to analyze pHsp70A-GLuc-derived *G. pectorale *transformants by measuring luciferase activities in heat-shocked and non-heat-shocked organisms. Transgene expression was induced by shifting the temperature of the culture from 23 to 36°C for 1 h. After a 1 h recovery phase at 23°C, luciferase activity was assayed at 20°C and the induction factors were calculated by comparison with non-heat-shocked samples. For comparison, induction factors were also calculated from pPsaD-GLuc-derived transformants, in which the luciferase gene is driven by the *psaD *promoter. The analyses show that heat shock induces luciferase expression to a much greater extent in those transformants in which the luciferase gene is driven by the *hsp*70A heat-shock promoter than it does in those in which the gene is driven by the *psaD *promoter (Figure 8C). Thus, the *C. reinhardtii hsp*70A heat-shock promoter is also inducible when utilized in *G. pectorale*.

### Long term stability of DNA integration and gene expression

For a variety of functional analyses, it is advantageous to have stable transformants that express the corresponding transgene for as long as possible. Forty independent *Gonium *transformants were propagated in medium that contained the antibiotic paromomycin (1 μg/ml) for two years (or longer), which corresponds to over 1400 colony generations. During this period, none of the transformant clones was lost. After this period, PCR using genomic DNA of these transformants as a template still yielded the expected 422 bp fragment of the *aphVIII *gene (data not shown).

In a parallel experiment, 25 independent transformants were kept in medium containing the antibiotic paromomycin (1 μg/ml) for ~200 colony generations, then propagated for ~200 colony generations in antibiotic-free medium without selective pressure, and finally cultivated again in medium containing paromomycin (1 μg/ml) for ~200 colony generations. Just as in the experiment under permanent selective pressure, all transformant clones survived this procedure, and the *aphVIII *gene was still detectable by genomic PCR.

The long term stability of co-transformed genes in 20 luciferase expressing transformants was also examined. Five months, or ~300 colony generations, after transformation with the selectable marker plasmid pPmr3 and plasmids pHsp70A-GLuc (10 co-transformants) or pPsaD-GLuc (10 co-transformants), the *luc *gene was still detectable by PCR in all the initially identified co-transformants. In addition, bioluminescence assays showed that luciferase activity was unchanged after this period.

## Discussion

Using the *aphVIII *gene of *S. rimosus *and a hybrid *hsp*70A/*rbc*S promoter along with an *rbc*S 3'-UTR from either *C. reinhardtii *or *V. carteri*, we have demonstrated that wild type *G. pectorale *strains can be transformed into paromomycin-resistant strains by particle gun bombardment. Since there was no information with regard to activity from any homologous *Gonium *promoter, we used heterologous promoters and were able to show their activity in *G. pectorale*. Although the frequency of transformation was low, the transformation process was reproducible. In addition, co-transformants were recovered with a high frequency, and transformants were stable for hundreds of colony generations under selective and nonselective conditions. Therefore, nuclear transformation of *G. pectorale *using hybrid, heterologous genes as selectable markers is feasible.

In *Gonium*, the frequency of transformation was estimated to be ~6.6 × 10^-7 ^or ~1.1 × 10^-7 ^per cell in particle bombardment experiments, depending on the plasmid used. In the related multicellular species *V. carteri *the mean frequency of stable transformants among the survivors of the particle bombardment was 2.5 × 10^-5 ^[6]. However, the frequency presumably would have been much lower if the calculation would have been per bombarded reproductive cell. In the unicellular relative *C. reinhardtii*, a transformation rate of 1.3 – 1.9 × 10^-7 ^per recipient cell was calculated when cell wall-deficient cells were treated using a glass bead transformation protocol [29]. In *Gonium*, the estimated efficiency of transformation was based on two assumptions, which were that all the cells that were used in a transformation experiment were bombarded by microprojectiles and that only a maximum of one transformant arose per cultivation flask. In reality, only a fraction of the cells are hit by a microprojectile. Most cells lie outside the target zone, many cells are covered by other cells, others are not hit even though they are within the target zone, and quite a few cells get lost during handling. In addition, it is very likely that more than one transformant occasionally arose per flask, but we counted and propagated only one transformant if a population of cells grew in a culture flask after particle bombardment under selective pressure. Due to these reasons, the efficiency of transformation, which is considered to be the number of independently transformed cells per total number of treated wild type cells, is probably higher than the estimated number given above, but it is not possible to determine the exact number of treated cells if a particle gun is used for transformation. The efficiency of transformation should not be a limiting factor for researchers because *Gonium *is a tiny organism that grows to high densities, and the cultivation of millions of colonies requires minimal expenses.

The cause of the better yields of *Gonium *transformation with the plasmid pPmr3 with its flanking sequences from *V. carteri *when compared with the plasmid paphG with its flanking sequences from *C. reinhardtii *and its 16 repeated cassettes remains unclear. Possible reasons might be that the *Volvox*-derived hybrid promoter in pPmr3 is more similar to *Gonium *promoters than the *Chlamydomonas*-derived hybrid promoter in paphG or that the uptake and integration of the 31.4 kb plasmid paphG were not as efficient as that of the 5.1 kb plasmid pPmr3.

For practical uses, the co-transformation rate is of special interest because it is more convenient to combine a known selectable marker plasmid and an unselectable plasmid containing the gene of interest, instead of constructing a single, large plasmid with unselectable and selectable genes. In *Gonium*, the co-transformation frequency was estimated to be ~30–50%, which means that ~3–5 co-transformants can be recovered from a single bombardment session of the type described in the Methods section. In *V. carteri*, frequencies of 10–60% [19] and 40–80% [6] were reported for two different transformation systems. In *C. reinhardtii*, frequencies of ~50% [32] or up to 80% [33] were achieved. These data show that co-transformation frequencies are quite high in *Gonium *and other volvocine algae, and, thus, co-transformation can be used routinely in these species.

We also show that the co-transformed genes were integrated into the genome and that they were also transcribed. We were not able to show protein function or protein expression in transformants that were generated using the plasmids ptubar4 and pHsp-HA, which should have made arylsulfatase and the tagged heat shock protein 70A, respectively. Possible reasons why the arylsulfatase or *hsp*70A genes fail to produce a detectable amount of (functional) protein are: 1) there are codons within these genes that are rarely or not used in *Gonium*; 2) the heterologous proteins are improperly folded, which leads not only to a lack of activity but also to quick protein degradation; 3) *Gonium *detects expression-limiting regulatory elements in the heterologous coding sequences [34]; 4) at least one intron/exon boundary within these heterologous genes is not easily recognized in the *Gonium *nucleus, which leads to a frameshift and a wrong/truncated polypeptide chain that is quickly degraded; 5) *Gonium *recognizes an additional intron/exon boundary in these heterologous genes, which also leads to a wrong/truncated polypeptide chain that is quickly degraded; 6) the arylsulfatase needs a posttranslational modification for activity [35], which is not added in *Gonium*.

However, transformants that were generated using the plasmids pPsaD-GLuc and pHsp70A-GLuc showed functional expression of luciferase through light emission, which is easily detected in bioluminescence assays. Therefore, the heterologous luciferase gene is a suitable reporter gene in *Gonium*.

## Conclusion

The availability of a transformation system that is based on a dominant selectable marker now makes extensive genetic engineering of *Gonium *possible. The strategy to use a bacterial antibiotic resistance gene and flanking sequences from close relatives might also be of interest for those researchers seeking to transform species without sequenced genomes but with sequenced relatives. The existence of a transformation system for *Gonium *also allows for more detailed studies of the molecular evolution of genes that regulate cellular differentiation, morphogenesis, and extracellular matrix biogenesis. This can be done by manipulating and comparing volvocine species with increasing organismal complexity, such as *Chlamydomonas*,* Gonium*, and *Volvox*, via expression of homologous, heterologous, artificial, chimeric (GFP-tagged), or otherwise modified genes.

## Methods

### Strains and culture conditions

The wild type *Gonium pectorale *Müller strains SAG 12.85, NIES-1710, and CCAP 32/14 were obtained from the Culture Collection of Algae at the University of Göttingen (SAG), Germany [36], the Microbial Culture Collection at the National Institute for Environmental Studies (NIES) (Tsukuba, Japan) and the Culture Centre of Algae and Protozoa (CCAP) (Ambleside, Scotland), respectively. Cultures were grown in Jaworski's Medium (JM) [37] at 23°C or 29°C in an 8 h dark/16 h light (~10,000 lux) cycle. Cultures were grown in 10 ml glass tubes with caps that allow for gas exchange or in 50 ml and 300 ml Erlenmeyer flasks, which were aerated via Pasteur pipettes with 40 cm^3 ^and 55 cm^3 ^sterile air/min, respectively. Transgenic strains that express the *aphVIII *gene were grown in JM in the presence of 1 μg paromomycin/ml (paromomycin sulfate, Sigma-Aldrich, St. Louis, MO).

### Transformation vectors

The plasmid pPmr3 contains the 0.8 kb *S. rimosus aphVIII *gene, which confers resistance to paromomycin, a *V. carteri hsp*70A-*rbc*S3 hybrid promoter (0.5 kb and 0.27 kb of upstream sequences), and a 3'-UTR from the *V. carteri rbc*S3 gene (0.53 kb of downstream sequence), and the total size of plasmid pPmr3 is 5.1 kb, which includes the pBluescript II vector backbone [21]. The plasmid paphG contains the 0.8 kb *S. rimosus aphVIII *gene, a *C. reinhardtii hsp*70A-*rbc*S2 hybrid promoter (0.26 kb and 0.22 kb of upstream sequences), intron 1 (0.15 kb) of the *C. reinhardtii rbc*S2 gene 42 bp upstream of the translation start codon, and a 3'-UTR of the *C. reinhardtii rbc*S2 gene (0.22 kb of downstream sequence), and the plasmid paphG contains sixteen repeats of this hybrid gene in the same orientation, which results in a 28.4 kb insert. The total size of plasmid paphG is 31.4 kb, which includes the pBluescript II vector backbone [22]. The plasmid ptubar4 contains the 7.8 kb *V. carteri *arylsulfatase (*ars*) gene, a *V. carteri *β2-tubulin promoter (0.5 kb of upstream sequence), and a *V. carteri *arylsulfatase 3'-UTR (2.3 kb of downstream sequence), and the total size of plasmid ptubar4 is 13.2 kb, which includes the pUC18 vector backbone [30]. The plasmid pHsp-HA contains the 3.2 kb *V. carteri hsp*70A gene with its own promoter (2.5 kb of upstream sequence) and its own 3'-UTR (0.75 kb of downstream sequence), and the coding sequence is tagged with a sequence coding for the HA-epitope. The total size of plasmid pHsp-HA is 9.4 kb, which includes the pBluescript II vector backbone [25]. The plasmid pPsaD-GLuc contains the 0.57 kb luciferase (*luc*) gene from *G. princeps*, which was engineered to match the codon usage in *C. reinhardtii*, a *C. reinhardtii psaD *promoter (0.8 kb of upstream sequence), and a *C. reinhardtii psaD *3'-UTR (0.55 kb of downstream sequence). The total size of plasmid pPsaD-GLuc is 5.0 kb, which includes the pBluescript II vector backbone [27]. The plasmid pHsp70A-GLuc contains the 0.57 kb luciferase (*luc*) gene from *G. princeps *(codon-optimized for *C. reinhardtii*) fused to a 0.8 kb DNA fragment that contains the first three exons of the *hsp*70B gene of *C. reinhardtii*, and the hybrid gene is driven by the *C. reinhardtii hsp*70A promoter (0.26 kb of upstream sequence) and the 3'-UTR comes from the *C. reinhardtii rbc*S2 gene (0.22 kb of downstream sequence). The total size of plasmid pHsp70A-GLuc is 4.9 kb, which includes the pBluescript II vector backbone [27].

### Preparation of plasmid DNA

Plasmid DNA was purified routinely using the E.Z.N.A.^® ^Plasmid Mini Kit II (Peqlab, Erlangen, Germany). Large plasmids (paphG) were purified from 50–100 ml *E. coli *cultures as described [38], but the anion exchange column step was omitted. The obtained plasmid DNA was further purified using the E.Z.N.A.^® ^Cycle Pure Kit (Peqlab).

### Coating of microprojectiles

For particle gun transformation (most successful combination of parameters as provided in Table 2), gold microprojectiles of 0.6 μm in diameter (Bio-Rad, Hercules, CA) were coated with the required plasmids. To that end, ~3 mg gold microprojectiles in 50 μl H_2_O were quickly mixed with 5 μg DNA of the circular selectable marker plasmid (concentration > 0.4 μg/μl), 5 μg DNA of the circular co-bombarded plasmid (if applicable), 50 μl 2.5 M CaCl_2_, and 20 μl 0.1 M spermidine (Sigma-Aldrich). Mixing was sustained for 30 min at 4°C. After the addition of 200 μl EtOH at room temperature, the suspension was centrifuged for 2–3 s at ~5000 g. The pellet was washed three times with 100 μl EtOH (at -20°C) and centrifuged for 2–3 s at ~5000 g. Finally, the DNA-coated particles were resuspended in 60 μl EtOH and kept at 4°C for use within 3 h.

### Determination of cell concentration

In *G. pectorale *the number of cells per colony varies. Therefore, we refer to "cells/ml" rather than "colonies/ml". Cell concentration was determined using a hemacytometer with Neubauer ruling.

### Stable nuclear transformation by particle gun

One hundred fifty milliliters of a logarithmically growing *G. pectorale *culture that contained ~6 × 10^4 ^cells/ml was harvested by centrifugation (800 g, 8 min, swing-out rotor) and resuspended in a total volume of 12 ml JM. Two milliliters of the suspension was spread evenly on a cellulose acetate membrane filter with a pore size of 1.2 μm and a diameter of 47 mm (Whatman, London, UK), and the filter was placed on top of a stack of absorbent paper that soaked up all the excess liquid. Stable transformation of *Gonium *(most successful combination of parameters as provided in Table 2) was performed using a Biolistic^® ^PDS-1000/He (Bio-Rad) particle gun. One-sixth of the DNA-coated microprojectiles were spread on a macrocarrier (Bio-Rad), which was placed in a macrocarrier holder (Bio-Rad). The distance between macrocarrier and stopping screen (Bio-Rad) was set to 8 mm. The helium pressure was defined by rupture disks with a burst pressure of 1100 psi (Bio-Rad). The gap between rupture disk and macrocarrier was adjusted to 7 mm. The membrane filter with its layer of *G. pectorale *was positioned in the bombardment chamber, the distance between the stopping screen and target cells was adjusted to 6 cm, and the chamber was partly evacuated to 27 inch Hg. After particle bombardment, the colonies were washed off from the membrane filter with JM. The procedure was repeated five times, and the colonies from the six bombardments were pooled and evenly distributed among ten 50 ml Erlenmeyer flasks that contained a final volume of ~35 ml JM each. Bombarded colonies were incubated under standard conditions for 48 h, and then 1 μg paromomycin/ml was added. Within 24 h, non-transformed cells died, which resulted in a clarification of the medium. After another 9–16 days of incubation in the presence of the antibiotic, greening of a flask showed the initial presence of at least one paromomycin-resistant cell that led to a population of transformants. No more than one transformant per flask was computed.

### Re-isolation of transformants

For detailed analyses, transformants were re-isolated to ensure uniform genetic condition. For this, a serial dilution of an exponentially growing *Gonium *culture was performed in a Terasaki plate (Nunc™ MicroWell™ MiniTrays; Thermo Fisher Scientific, Langenselbold, Germany), which was filled with 10 μl JM medium per well. Under microscopic control, a single *Gonium *colony was finally transferred into a standard glass tube with JM medium containing 1 μg paromomycin/ml and incubated under standard conditions.

### Paromomycin-resistance assay

Transformants or wild type strains were transferred into glass tubes with increasing concentrations of paromomycin in JM. At the beginning of the assay, each tube contained ~12,000 healthy cells in a total volume of 10 ml. Incubation under standard conditions continued for eight days. Subsequently, the tubes were analyzed for either viable, green cells/colonies or cell lysis with some white remains of dead cells/colonies.

### Primer design

Oligonucleotide primers were designed using the primer analysis software Oligo 6 (Molecular Biology Insights, Cascade, CO), DNASIS™ (version 7.00; Hitachi Software Engineering, San Francisco, CA), and Primer Express^® ^(Applied Biosystems, Foster City, CA).

### Isolation of genomic DNA

Thirty-five milliliters of a logarithmically growing culture was harvested by centrifugation (3500 g, 10 min). The pellet, which had a wet weight of ~80 mg, was washed twice with H_2_O, centrifuged 2×, resuspended in H_2_O, and frozen in liquid nitrogen. Frozen samples were pulverized in a mortar. After homogenization, the sample was warmed to 65°C, and lysis buffer (Qiagen, Hilden, Germany) that contained RNase A1 was added. Genomic DNA was isolated using the spin columns of the DNeasy^® ^Plant Mini Kit (Qiagen).

Larger amounts of genomic DNA were prepared by conventional methods [39], using tris-saturated phenol (Roti^®^-phenol; Roth, Karlsruhe, Germany).

### Genomic PCR

Genomic PCR was carried out in a total volume of 50 μl, which contained ~100 ng of genomic DNA, 300 nM of each primer, 0.2 mM dNTP mix, 1.5 mM MgCl_2_, and 2.6 units of Expand High Fidelity enzyme mix in 1× Expand High Fidelity buffer (Roche Applied Science, Basel, Switzerland). PCR was performed on a T3 Thermocycler PCR system (Biometra, Göttingen, Germany) using the following conditions: 40 cycles of 94°C for 20 s, 55°C for 30 s, and 72°C for 45 s and a final extension was at 72°C for 10 min. The PCR products were cloned and sequenced.

### Southern blotting

After restriction enzyme digest, genomic DNA fragments were separated on 1% agarose gels, vacuum transferred to nylon membranes (Hybond-N^®^; Amersham Biosciences, Little Chalfont, UK), and fixed to the membrane by baking for 30 min at 120°C using standard protocols [39]. A 282 bp fragment of the *aphVIII *coding region was amplified by PCR (Expand High Fidelity Plus PCR System; Roche Applied Science) and simultaneously labeled using a digoxigenin DNA labeling mix (Roche Applied Science). A 343 bp fragment of the coding region of the luciferase (*luc*) gene from *G. princeps *(codon-optimized for *C. reinhardtii*) was amplified in the same way. Pre-hybridization at 52°C, hybridization at 52°C, and washing steps were carried out in standard solutions (Roche Applied Science). Detection of the hybridizing bands was done by using an anti-digoxigenin-alkaline phosphatase conjugate (1:7500 dilution) and the chemiluminescent substrate CDP Star^®^, in accordance with the instructions of the supplier of the chemiluminescence reagent (Roche Applied Science). Chemiluminescence-sensitive films (Retina XBA; Fotochemische Werke, Berlin, Germany) were subsequently exposed to the membranes for 2–15 min.

### Isolation of total RNA

Total RNA was isolated from ~9 × 10^6 ^*Gonium *cells using the membrane-based SV Total RNA Isolation System (Promega, Madison, WI). RNA quantification and purity checks were done by agarose gel electrophoresis and by measuring absorption at 260 and 280 nm with an Ultrospec™ 2100 pro UV/Visible Spectrophotometer (GE Healthcare, Uppsala, Sweden).

### Reverse Transcription (RT)-PCR

First strand cDNA synthesis was performed using 1 μg total RNA and Moloney murine leukemia virus (MMLV) reverse transcriptase lacking ribonuclease H activity (H minus), according to the manufacturer's instructions (Promega). Subsequent PCR was carried out using the Mid Range PCR system, according to the instructions provided by the vendor (Peqlab). PCR was performed on a T3 Thermocycler PCR system (Biometra) using the following cycling conditions: 40 cycles of 94°C for 20 s, 55°C for 30 s, and 68°C for 45 s and a final extension at 68°C for 10 min. The RT-PCR products were cloned and sequenced.

### Western blot analysis

Eight hundred milliliters of a logarithmically growing culture containing ~5 × 10^7 ^cells was harvested by centrifugation (3500 g, 8 min, swing-out rotor), washed with 20 mM phosphate buffer (pH 7.4), and disrupted using a Sonopuls™ HD2070 sonicator (Bandelin Electronic, Berlin, Germany). The lysate was cleared by centrifugation (86,000 g, 90 min), passed through a Centricon^® ^100 column (Millipore, Bedford, MA), concentrated on a Centricon^® ^10 column (Millipore), and used for western blot analysis. Samples were separated on a 10% standard SDS-polyacrylamide gel, electroblotted to a polyvinylidene fluoride membrane (0.45 μm; Millipore), and probed using a purified polyclonal rabbit anti-AphVIII antibody at 1:100 dilution [20,21]. The secondary antibody was a horseradish peroxidase-linked anti-rabbit-IgG at 1:10,000 dilution (Bio-Rad). Signals were visualized by using the luminol-based chemiluminescent substrate Lumiglo^® ^(Cell Signaling Technology, Danvers, MA) and Hyperfilm™ ECL films (Amersham Biosciences).

### Luciferase assays

For assays on light-sensitive films, a *Gonium *culture (50 ml) with 3–6 × 10^6 ^cells/ml was centrifuged, resuspended in 850 μl assay buffer [0.1 M K_2_HPO_4 _(pH 7.6), 0.5 M NaCl, 1 mM EDTA] and cells were disrupted using a Sonopuls™ HD2070 sonicator (Bandelin Electronic) and the lysate was transferred to a 24-well plate. After addition of 150 μl 0.05 mM coelenterazine (Fluka, Neu-Ulm, Germany) in assay buffer, the 24-well plate was exposed to a chemiluminescence-sensitive film (Retina XBA; Fotochemische Werke) for 30 s at 20°C [40].

Quantitation of bioluminescence was performed as described by Shao and Bock [27]. For it, 5 ml of a *Gonium *culture, which has been grown at 23°C to a density of 3–6 × 10^6 ^cells/ml, was centrifuged, resuspended in 300 μl sample buffer [1.5 mM Tris-HCl (pH 7.8), 1 mM EDTA], and frozen at -20°C for at least 20 min. After thawing, 20 μl samples were added to 125 μl of the assay buffer [0.1 M K_2_HPO_4 _(pH 7.6), 0.5 M NaCl, 1 mM EDTA]. Following incubation for 15 min at 20°C in the dark, samples were transferred to clear polystyrene vials (Sarstedt, Nümbrecht, Germany), 50 μl 0.01 mM coelenterazine was added, and bioluminescence was assayed at 20°C using a MiniLumat LB9506 luminometer (Berthold, Bad Wildbad, Germany). The luminescence was recorded as relative light units.

For analysis of induction of luciferase activity in heat-shocked transformants, organisms were subject to a temperature shift from 23 to 36°C for 1 h, because in preliminary tests, shifts to 36° resulted in the strongest induction in comparison to lower or higher temperatures (data not shown). After a 1 h recovery phase at 23°C, cells were lysed by freezing and thawing and luciferase activity was assayed at 20°C as described above [27]. As a reference, non-heat-shocked transformants were analyzed in the same way. The induction factors were calculated by comparison of heat-shocked with non-heat-shocked samples.

### Phylogenetic analysis

Alignment of sequences was done using the MUltiple Sequence Comparison by Log-Expectation program (MUSCLE) [41]. Minor manual optimization of alignments, trimming, and management of multi-aligned data was done with BioEdit v7.0.9 [42]. Alignments were illustrated using GeneDoc 2.6 [43]. The Needleman-Wunsch global alignment algorithm [44] from the European Molecular Biology Open Software Suite (EMBOSS) was used for the comparison of two sequences [45]. Unrooted consensus trees were calculated using the PHYLogeny Inference Package (PHYLIP) [46]. For each consensus tree, 10000 bootstrap resamplings of multi-aligned sequences were generated with Seqboot, distance matrices were computed with Dnadist, trees were constructed using the neighbor-joining method [47] as implemented in Neighbor, and finally a consensus tree was built using Consense. Phylogenetic trees were drawn with TreeView [48].

### GenBank accession numbers

The novel sequences that are described in this study have been deposited under the following accession numbers:

*Gonium pectorale *SAG 12.85: *rbc*L [GenBank: FJ793553], *psa*A [GenBank: FJ793556], *psa*B [GenBank: FJ793559], ITS [GenBank: FJ793562]; *Gonium pectorale *CCAP 32/14: *rbc*L [GenBank: FJ793554], *psa*A [GenBank: FJ793557], *psa*B [GenBank: FJ793560], ITS [GenBank: FJ793563]; *Gonium pectorale *NIES-1710: *rbc*L [GenBank: FJ793555], *psa*A [GenBank: FJ793558], *psa*B [GenBank: FJ793561], ITS [GenBank: FJ793564].

The accession numbers of other cited sequences are:

*Gonium pectorale *NIES-569: *rbc*L [GenBank: D63437], *psa*A [GenBank: AB044242], *psa*B [GenBank: AB044463]; *Gonium pectorale *UTEX 2570: ITS [GenBank: AF054425]; *Gonium pectorale *AWCAf2–3: ITS [GenBank: AF054431]; *Gonium pectorale *AWC-Laos: ITS [GenBank: AF182429]; *Gonium pectorale *Coleman 16-1: ITS [GenBank: U66969]; *Gonium pectorale *UTEX 2075: ITS [GenBank: AF054434]; *Gonium pectorale *UTEX 2581: ITS [GenBank: AF054433]; *Gonium octonarium *GO-LC-1+: *rbc*L [GenBank: D63436], *psa*A [GenBank: AB044241], *psa*B [GenBank: AB044462]; *Gonium octonarium *UTEX 842: ITS [GenBank: U66968]; *Gonium quadratum *NIES-653: *rbc*L [GenBank: D63438], *psa*A [GenBank: AB044243], *psa*B [GenBank: AB044464]; *Gonium quadratum *AWC-Cal3-3: ITS [GenBank: AF182430]; *Gonium quadratum *AWC-Cat: ITS [GenBank: AF182431]; *Gonium multicoccum *UTEX 2580: *rbc*L [GenBank: D63435], *psa*A [GenBank: AB044240], *psa*B [GenBank: AB044461]; *Gonium multicoccum *UTEX 783: ITS [GenBank: U66967]; *Gonium viridistellatum *UTEX 2519: *rbc*L [GenBank: D86831], *psa*A [GenBank: AB044244], *psa*B [GenBank: AB044465]; *Gonium viridistellatum *UTEX 2520: ITS [GenBank: AF182432]; *Tetrabaena socialis *(= *Gonium sociale*) NIES-571: *rbc*L [GenBank: D63443], *psa*A [GenBank: AB044415], *psa*B [GenBank: AB044466]; *Tetrabaena socialis *(= *Gonium sociale*) UTEX 14: ITS [GenBank: U66976]; *Basichlamys sacculifera *(= *Gonium sacculiferum*) NIES-566: *rbc*L [GenBank: D63430], *psa*A [GenBank: AB044416], *psa*B [GenBank: AB044467, AB044468]; *Basichlamys sacculifera *(= *Gonium sacculiferum*) UTEX 822: ITS [GenBank: U66972]; *Astrephomene gubernaculifera *NIES-418: *rbc*L [GenBank: D63428], *psa*A [GenBank: AB044234], *psa*B [GenBank: AB044458]; *Astrephomene gubernaculifera *UTEX 1393: ITS [GenBank: AF054422]; *Astrephomene perforata *NIES-564: *rbc*L [GenBank: D63429], *psa*A [GenBank: AB044238], *psa*B [GenBank: AB044460]; *Astrephomene perforata *UTEX 2475: ITS [GenBank: U66939]; *Pandorina morum *NIES-574: *rbc*L [GenBank: D63442], *psa*A [GenBank: AB044226], *psa*B [GenBank: AB044452]; *Pandorina morum *Poona: ITS [GenBank: AF182433]; *Eudorina unicocca *UTEX 1215: *rbc*L [GenBank: D63434], *psa*A [GenBank: AB044209], *psa*B [GenBank: AB044440]; *Eudorina elegans *NIES-456: *rbc*L [GenBank: D63432], *psa*A [GenBank: AB044199], *psa*B [GenBank: AB044435]; *Pleodorina californica *UTEX 809: *rbc*L [GenBank: D63439], *psa*A [GenBank: AB044192], *psa*B [GenBank: AB044430]; *Volvox aureus *NIES-541: *rbc*L [GenBank: D63445], *psa*A [GenBank: AB044182], *psa*B [GenBank: AB044424]; *Volvox carteri *NIES-732: *rbc*L [GenBank: D63446], *psa*A [GenBank: AB044185], *psa*B [GenBank: AB044425]; *Volvox globator *UTEX 955: *rbc*L [GenBank: D86836], *psa*A [GenBank: AB044187], *psa*B [GenBank: AB044428]; *Chlamydomonas reinhardtii *137C: *rbc*L [GenBank: J01399], *psa*A [GenBank: AB044419], *psa*B [GenBank: AB044470]; plasmid pPmr3 [GenBank: AY429514].

## Abbreviations

*aphVIII*: aminoglycoside 3'-phosphotransferase VIII gene; *ars*: arylsulfatase gene; gPCR: genomic PCR; *hsp*70A: heat shock protein 70A gene; ITS: internal transcribed spacer; JM: Jaworski's Medium; MMLV: Moloney murine leukemia virus; PCR: polymerase chain reaction; *psa*A: photosystem I P700 chlorophyll a apoprotein A1 gene; *psa*B: photosystem I P700 chlorophyll a apoprotein A2 gene; *psaD*: photosystem I reaction center subunit II (chloroplastic) gene; *rbc*L: ribulose-1,5-bisphosphate carboxylase (large subunit) gene; *rbc*S: ribulose-1,5-bisphosphate carboxylase (small subunit) gene; rRNA: ribosomal RNA; RT-PCR: reverse transcription PCR; UTR: untranslated region.

## Authors' contributions

KL conducted the experiments and analyzed the data. AH (corresponding author) conceived and coordinated the study, critically evaluated the data, and wrote the manuscript. All authors read and approved the final manuscript.

## Supplementary Material

Additional file 1 **Description of the phylogenetic analysis of utilized *Gonium pectorale *strains**. The identity of the utilized *Gonium pectorale *strains SAG 12.85, CCAP 32/14 and NIES-1710 was verified in a phylogenetic analysis. Therefore, we cloned and sequenced certain DNA fragments that have been used in phylogenetic analyses of other volvocine algae. These include fragments of chloroplast genes encoding photosystem I P700 chlorophyll a apoprotein A1 (*psa*A), photosystem I P700 chlorophyll a apoprotein A2 (*psa*B) and ribulose bisphosphate carboxylase (*rbc*L), as well as the internal transcribed spacer sequences, ITS 1 and ITS 2, that flank the 5.8S ribosomal RNA (rRNA) nuclear gene. Click here for file

Additional file 2 **Sequence alignment of *psa*A cDNA fragments from several volvocine species.** Click here for file

Additional file 3 **Sequence alignment of *psa*B cDNA fragments from several volvocine species.** Click here for file

Additional file 4 **Sequence alignment of *rbc*L cDNA fragments from several volvocine species.** Click here for file

Additional file 5 **Sequence alignment of ITS sequences flanking the 5.8S rRNA gene from several volvocine species.** Click here for file

Additional file 6 **Sequence comparison of *psa*A, *psa*B and *rbc*L from several volvocine species.** Click here for file

Additional file 7 **Sequence comparison of ITS sequences flanking the 5.8S rRNA gene from several volvocine species** Click here for file

Additional file 8 **Phylogeny based on *psa*A cDNA fragments from several volvocine species**. Relationships among *psa*A cDNA fragments from several volvocine species. The unrooted tree was calculated using the neighbor-joining method of PHYLIP. Numbers indicate bootstrap analysis values obtained using 10000 resampled data sets. The analysis is based on the alignment given in Additional File 2. All *Gonium pectorale *strains are highlighted in light blue. *Gonium pectorale *strains used in this study are indicated by a dark blue arrow. Click here for file

Additional file 9 **Phylogeny based on *psa*B cDNA fragments from several volvocine species**. Relationships among *psa*B cDNA fragments from several volvocine species. The unrooted tree was calculated using the neighbor-joining method of PHYLIP. Numbers indicate bootstrap analysis values obtained using 10000 resampled data sets. The analysis is based on the alignment given in Additional File 3. All *Gonium pectorale *strains are highlighted in light blue. *Gonium pectorale *strains used in this study are indicated by a dark blue arrow. Click here for file

Additional file 10 **Phylogeny based on *rbc*L cDNA fragments from several volvocine species**. Relationships among *rbc*L cDNA fragments from several volvocine species. The unrooted tree was calculated using the neighbor-joining method of PHYLIP. Numbers indicate bootstrap analysis values obtained using 10000 resampled data sets. The analysis is based on the alignment given in Additional File 4. All *Gonium pectorale *strains are highlighted in light blue. *Gonium pectorale *strains used in this study are indicated by a dark blue arrow. Click here for file

Additional file 11 **Phylogeny based on a combined data set of *psa*A, *psa*B and *rbc*L cDNA fragments from several volvocine species**. Relationships within a combined data set generated from the *psa*A, *psa*B and *rbc*L sequences from several volvocine species. The unrooted tree was calculated using the neighbor-joining method of PHYLIP. Numbers indicate bootstrap analysis values obtained using 30000 resampled data sets. The analysis is based on the alignments given in Additional Files 2, 3 and 4. All *Gonium pectorale *strains are highlighted in light blue. *Gonium pectorale *strains used in this study are indicated by a dark blue arrow. Click here for file

Additional file 12 **Phylogeny based on ITS 1, ITS 2 and 5.8S rRNA sequences from several volvocine species**. Relationships among ITS 1/5.8S rRNA/ITS 2 sequences from several volvocine species. The unrooted tree was calculated using the neighbor-joining method of PHYLIP. Numbers indicate bootstrap analysis values obtained using 10000 resampled data sets. The analysis is based on the alignment given in Additional File 5. All *Gonium pectorale *strains are highlighted in light blue. *Gonium pectorale *strains used in this study are indicated by a dark blue arrow. Click here for file
