# Supplementary material for: Stable nuclear transformation of Gonium pectorale
Source: BMC Biotechnol. 2009 Jul 10;9:64. doi: 10.1186/1472-6750-9-64 (PMC2720962; doi:10.1186/1472-6750-9-64)
Supplement: Additional file 1 — Description of the phylogenetic analysis of utilized Gonium pectorale strains. The identity of the utilized Gonium pectorale strains SAG 12.85, CCAP 32/14 and NIES-1710 was verified in a phylogenetic analysis. Therefore, we cloned and sequenced certain DNA fragments that have been used in phylogenetic analyses of other volvocine algae. These include fragments of chloroplast genes encoding photosystem I P700 chlorophyll a apoprotein A1 (psaA), photosystem I P700 chlorophyll a apoprotein A2 (psaB) and ribulose bisphosphate carboxylase (rbcL), as well as the internal transcribed spacer sequences, ITS 1 and ITS 2, that flank the 5.8S ribosomal RNA (rRNA) nuclear gene. [file 1472-6750-9-64-S1.pdf]

Description of the phylogenetic analysis of utilized *Gonium pectorale* strains

Our standard *Gonium pectorale* strain is SAG 12.85, which we have used for several years. This strain was originally collected by D. Albers in 1982 in a pond of the Botanical Garden at the University of Göttingen and was deposited at the Culture Collection of Algae at the University of Göttingen (SAG) (Schlösser, 1994). Details about how the species identification was performed are not available. Recently, there have been some doubts within the scientific community regarding the identity of *G. pectorale* strain SAG 12.85. Therefore, we checked the identity of SAG 12.85 along with two other wild type *G. pectorale* strains, NIES-1710 and CCAP 32/14, by cloning and sequencing of certain DNA fragments that have been used in phylogenetic analyses of other volvocine algae (Nozaki et al., 2000; Nozaki, 2003; Coleman et al., 1994; Mai and Coleman, 1997; Coleman and Mai, 1997; Coleman et al., 1998; Coleman, 1999). These include fragments of chloroplast genes encoding photosystem I P700 chlorophyll a apoprotein A1 (*psa*A), photosystem I P700 chlorophyll a apoprotein A2 (*psa*B) and ribulose bisphosphate carboxylase (*rbc*L) (Nozaki et al., 2000; Nozaki, 2003), as well as the internal transcribed spacer sequences, ITS 1 and ITS 2, that flank the 5.8S ribosomal RNA (rRNA) nuclear gene (Coleman et al., 1994; Mai and Coleman, 1997; Coleman and Mai, 1997; Coleman et al., 1998; Coleman, 1999). Oligonucleotide primers for PCR were designed based on multiple alignments of known sequences from other volvocine species/strains. The sequences of the obtained DNA fragments from SAG 12.85, CCAP 32/14, and NIES-1710 were aligned with previously known sequences from volvocine species/strains, and the alignments are shown in Additional Files 2 (*psa*A), 3 (*psa*B), 4 (*rbc*L) and 5 (ITS 1/5.8S rRNA/ITS 2). Sequence identities with several related sequences from other volvocine species/strains were calculated for each of the four DNA sequences (*psa*A, *psa*B, *rbc*L and ITS 1/5.8S rRNA/ITS 2). The results with *psa*A, *psa*B and *rbc*L are shown in Additional File 6, the results with ITS 1/5.8S rRNA/ITS 2 are given in Additional File 7. In these analyses, strains SAG 12.85, CCAP 32/14 and NIES-1710 always show the highest sequence identities with other *G. pectorale* strains for all four of the DNA sequences.

Based on the sequence alignments shown in Additional Files 2, 3, 4 and 5, also phylogenetic trees of volvocine algae have been calculated. The trees are shown in Additional Files 8 (*psa*A), 9 (*psa*B), 10 (*rbc*L), 11 (*psa*A, *psa*B and *rbc*L) and 12 (ITS 1/5.8S rRNA/ITS 2). The tree in Additional File 11 is a combined data set generated from the *psa*A, *psa*B and *rbc*L sequences.

In all these trees, the *G. pectorale* strains are located within the same branch as expected. There is only one exception: In the tree that is based on ITS 1/5.8S rRNA/ITS 2 sequences (Additional File 12) the strain AWC-Af2-3 is outside the *G. pectorale* branch. Therefore the

species identity of strain AWC-Af2-3 seems to be doubtful and needs to be rechecked in more detail; however, strain AWC-Af2-3 was not used in our transformation experiments.

In every tree all *G. pectorale* strains used for transformation experiments (SAG 12.85, CCAP 32/14, and NIES-1710) are located within the *G. pectorale* branch. Therefore, SAG 12.85 is clearly a *G. pectorale* strain and the same is true for the strains CCAP 32/14 and NIES-1710.

References

- Coleman AW: **Phylogenetic analysis of "Volvocaceae" for comparative genetic studies**. *Proc Natl Acad Sci USA* 1999, **96**:13892-13897.
- Coleman AW, Mai JC: **Ribosomal DNA ITS-1 and ITS-2 sequence comparisons as a tool for predicting genetic relatedness**. *J Mol Evol* 1997, **45**:168-177.
- Coleman AW, Preparata RM, Mehrotra B, Mai JC: **Derivation of the secondary structure of the ITS-1 transcript in Volvocales and its taxonomic correlations**. *Protist* 1998, **149**:135-146.
- Coleman AW, Suarez A, Goff LJ: **Molecular delineation of species and syngens in volvocacean green algae (Chlorophyta)**. *J Phycol* 1994, **30**:80-90.
- Mai JC, Coleman AW: **The internal transcribed spacer 2 exhibits a common secondary structure in green algae and flowering plants**. *J Mol Evol* 1997, **44**:258-271.
- Nozaki H: **Origin and evolution of the genera *Pleodorina* and *Volvox* (Volvocales)**. *Biologia (Bratisl)* 2003, **58**:425-431.
- Nozaki H, Misawa K, Kajita T, Kato M, Nohara S, Watanabe MM: **Origin and evolution of the colonial volvocales (Chlorophyceae) as inferred from multiple, chloroplast gene sequences**. *Mol Phylogenet Evol* 2000, **17**:256-268.
- Schlösser UG: **SAG-Sammlung von Algenkulturen at the University of Göttingen, Catalogue of Strains 1994**. *Bot Acta* 1994, **107**:111-186.
