# Supplementary material for: Stable nuclear transformation of Gonium pectorale
Source: BMC Biotechnol. 2009 Jul 10;9:64. doi: 10.1186/1472-6750-9-64 (PMC2720962; doi:10.1186/1472-6750-9-64)
Supplement: Additional file 2 — Sequence alignment of psaA cDNA fragments from several volvocine species. [file 1472-6750-9-64-S2.pdf]

# Sequence alignment of *psa*A cDNA fragments from several volvocine species

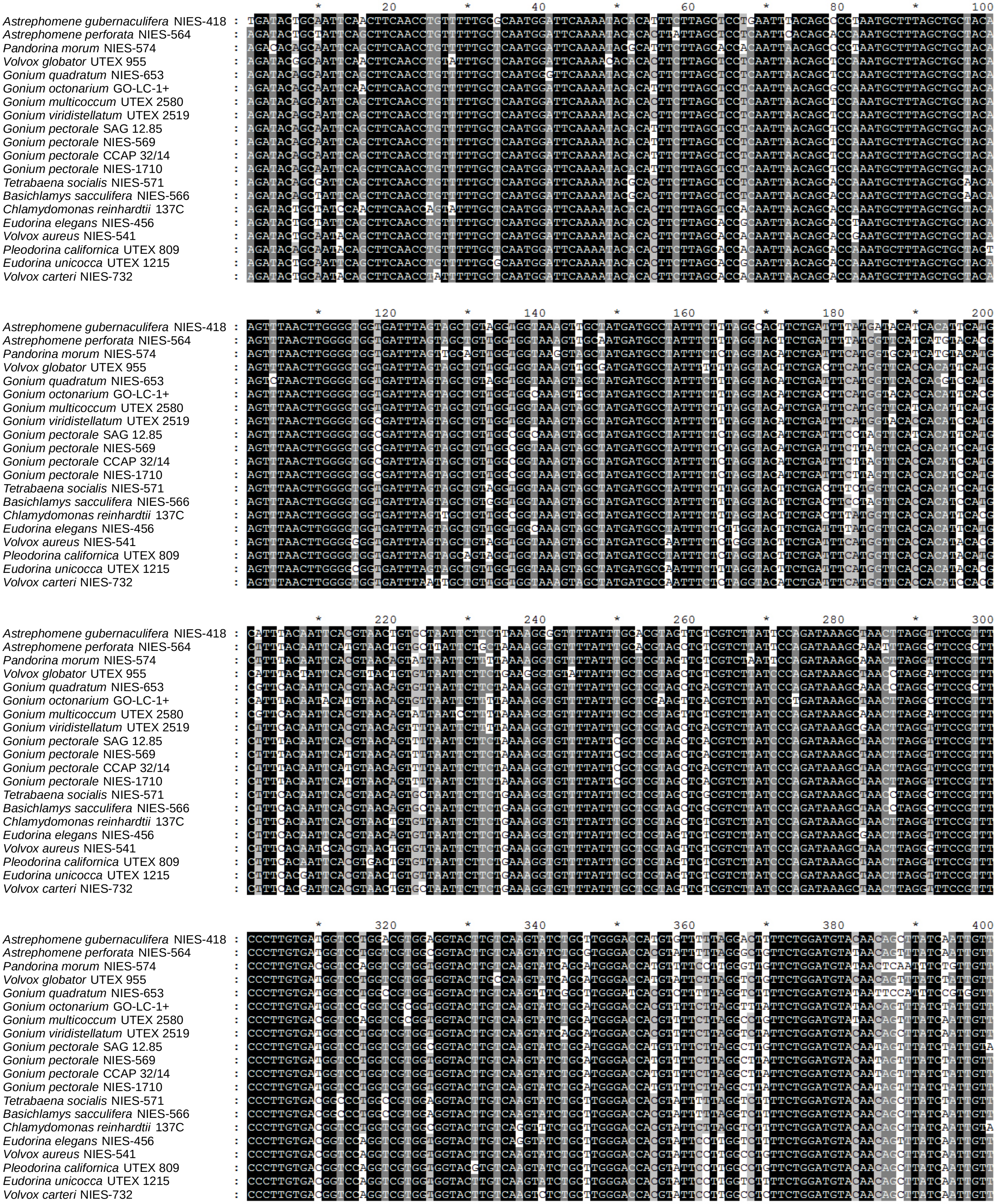

| | | * | | |
|---|---|---|---|---|
| *Astrephomene gubernaculifera* NIES-418 | : | ATTTTCCACTTCAGC | : | 415 |
| *Astrephomene perforata* NIES-564 | : | ATTTTCCACTTTAGT | : | 415 |
| *Pandorina morum* NIES-574 | : | ATTTTCCACTTTAGT | : | 415 |
| *Volvox globator* UTEX 955 | : | ATTTTCCACTTTAGT | : | 415 |
| *Gonium quadratum* NIES-653 | : | ATTTTCCACTTTAGC | : | 415 |
| *Gonium octonarium* GO-LC-1+ | : | ATTTTCCACTTCAGC | : | 415 |
| *Gonium multicoccum* UTEX 2580 | : | ATTTTCCACTTCAGC | : | 415 |
| *Gonium viridistellatum* UTEX 2519 | : | ATTTTCCACTTCAGC | : | 415 |
| *Gonium pectorale* SAG 12.85 | : | ATTTTCCACTTCAGC | : | 415 |
| *Gonium pectorale* NIES-569 | : | ATTTTCCACTTCAGT | : | 415 |
| *Gonium pectorale* CCAP 32/14 | : | ATTTTCCACTTCAGT | : | 415 |
| *Gonium pectorale* NIES-1710 | : | ATTTTCCACTTCAGT | : | 415 |
| *Tetrabaena socialis* NIES-571 | : | ATTTTCCACTTCAGC | : | 415 |
| *Basichlamys sacculifera* NIES-566 | : | ATTTTCCACTTTAGT | : | 415 |
| *Chlamydomonas reinhardtii* 137C | : | ATTTTCCACTTCAGC | : | 415 |
| *Eudorina elegans* NIES-456 | : | ATTTTCCACTTCAGC | : | 415 |
| *Volvox aureus* NIES-541 | : | ATTTTCCACTTTAGC | : | 415 |
| *Pleodorina californica* UTEX 809 | : | ATTTTCCACTTCAGC | : | 415 |
| *Eudorina unicocca* UTEX 1215 | : | ATTTTCCACTTCAGC | : | 415 |
| *Volvox carteri* NIES-732 | : | ATTTTCCACTTCAGC | : | 415 |

Alignment of sequences was done using the MUltiple Sequence Comparison by Log-Expectation program (MUSCLE) (Edgar, 2004). Conserved amino acid residues were shaded using GeneDoc 2.6 (Nicholas et al., 1997). White letters on black background: conserved in 100 percent of the sequences at the corresponding position; white letters on dark gray background: conserved in >80 percent of the sequences at the corresponding position; black letters on light gray background: conserved in >60 percent of the sequences at the corresponding position.

References


- Edgar RC: MUSCLE: multiple sequence alignment with high accuracy and high throughput. Nucleic Acids Res 2004, 32:1792-1797.
- Nicholas KB, Nicholas HB, Deerfield DW: GeneDoc: Analysis and visualization of genetic variation. Embnet News 1997, 4:14.
