# Supplementary material for: Stable nuclear transformation of Gonium pectorale
Source: BMC Biotechnol. 2009 Jul 10;9:64. doi: 10.1186/1472-6750-9-64 (PMC2720962; doi:10.1186/1472-6750-9-64)
Supplement: Additional file 4 — Sequence alignment of rbcL cDNA fragments from several volvocine species. [file 1472-6750-9-64-S4.pdf]

Sequence alignment of *rbc*L cDNA fragments from several volvocine species

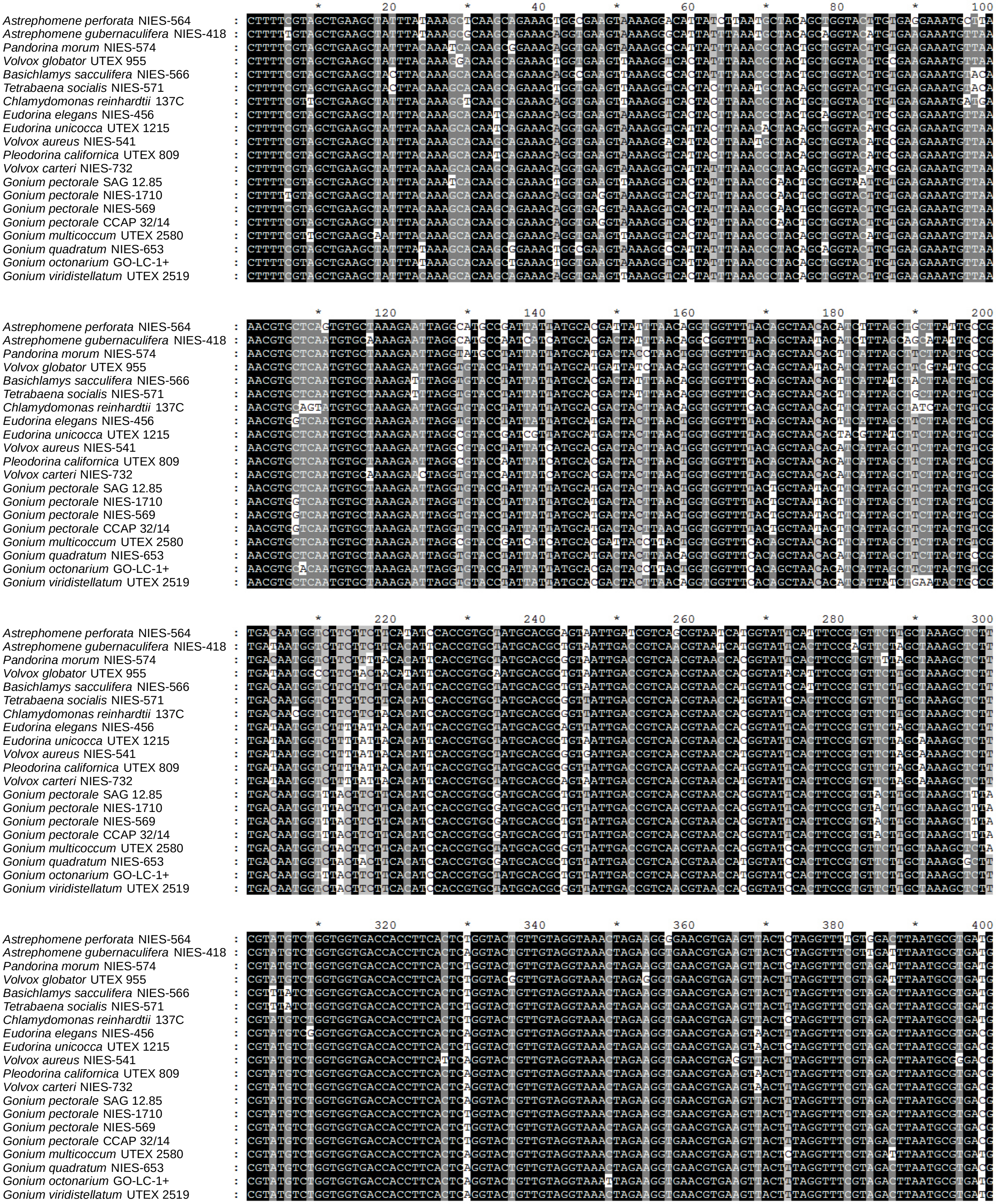

```
                                                          *        420         *        440         *
Astrephomene perforata NIES-564          : ACTATATTGAAAAAGACCGTAGCCGTGGTATTTACTTTACACAAAACTGGTGTTCTA : 457
Astrephomene gubernaculifera NIES-418    : ATTACATTGAAAAAGATCGTAGCCGTGGTATTTACTTTACACAAGACTGGTGTTCAA : 457
Pandorina morum NIES-574                 : ACTACGTTGAAAAAGACCGTAGCCGTGGTATTTACTTTACTCAAGACTGGTGTTCAA : 457
Volvox globator UTEX 955                 : ACTACATTGAAAAAGATCGTAGCCGTGGTATTTACTTTACTCAAGATTGGTGTTCAA : 457
Basichlamys sacculifera NIES-566         : ACTACGTAGAAAAAGATCGTAGCCGTGGTATTTATTTCACTCAAGATTGGTGTTCTA : 457
Tetrabaena socialis NIES-571             : ACTACGTTGAAAAAGATCGTAGCCGTGGTATTTATTTCACTCAAGACTGGTGTTCTA : 457
Chlamydomonas reinhardtii 137C           : ACTACGTTGAAAAAGACCGTAGCCGTGGTATTTACTTCACTCAAGACTGGTGTTCAA : 457
Eudorina elegans NIES-456                : ACTATATCGAAAAAGATCGTAGTCGTGGTATTTACTTCACTCAAGACTGGTGTTCAA : 457
Eudorina unicocca UTEX 1215              : ACTATGTTGAAAAAGATCGTAGCCGTGGTATTTACTTCACTCAAGACTGGTGTTCAA : 457
Volvox aureus NIES-541                   : ACTATATCGAAAAAGATCGTAGCCGTGGTATTTACTTTACGCAAAACTGGTGTTCAA : 457
Pleodorina californica UTEX 809          : ACTATATCGAAAAAGATCGTAGCCGTGGTATTTACTTCACGCAAGACTGGTGTTCAA : 457
Volvox carteri NIES-732                  : ACTATATCGAAAAAGATCGTAGCCGTGGTATTTACTTTACACAAGACTGGTGTTCAA : 457
Gonium pectorale SAG 12.85               : ACTATATTGAAAAAGATCGTAGCCGTGGTATTTACTTCACACAAGATTGGTGTTCAA : 457
Gonium pectorale NIES-1710               : ACTATATTGAAAAAGATCGTAGCCGTGGTATTTACTTCACACAAGATTGGTGTTCAA : 457
Gonium pectorale NIES-569                : ACTATATTGAAAAAGATCGTAGCCGTGGTATTTACTTCACACAAGATTGGTGTTCAA : 457
Gonium pectorale CCAP 32/14              : ACTATATTGAAAAAGATCGTAGCCGTGGTATTTACTTCACACAAGATTGGTGTTCAA : 457
Gonium multicoccum UTEX 2580             : ACTACATTGAAAAAGACCGTAGCCGTGGTATTTACTTCACTCAAGACTGGTGTTCAA : 457
Gonium quadratum NIES-653                : ACTACATTGAAAAAGACCGTAGCCGTGGTATTTACTTTACTCAAGATTGGTGTTCAA : 457
Gonium octonarium GO-LC-1+               : ACTACATTGAAAAAGATCGTAGCCGTGGTATTTACTTTACTCAAGATTGGTGTTCAA : 457
Gonium viridistellatum UTEX 2519         : ACTACATTGAAAAAGACCGTAGCCGTGGTATTTACTTTACTCAAGATTGGTGTTCAA : 457
```

Alignment of sequences was done using the MUltiple Sequence Comparison by Log-Expectation program (MUSCLE) (Edgar, 2004). Conserved amino acid residues were shaded using GeneDoc 2.6 (Nicholas et al., 1997). White letters on black background: conserved in 100 percent of the sequences at the corresponding position; white letters on dark gray background: conserved in >80 percent of the sequences at the corresponding position; black letters on light gray background: conserved in >60 percent of the sequences at the corresponding position.

References

- Edgar RC: MUSCLE: multiple sequence alignment with high accuracy and high throughput. Nucleic Acids Res 2004, 32:1792-1797.
- Nicholas KB, Nicholas HB, Deerfield DW: GeneDoc: Analysis and visualization of genetic variation. Embnet News 1997, 4:14.
