# Supplementary material for: Stable nuclear transformation of Gonium pectorale
Source: BMC Biotechnol. 2009 Jul 10;9:64. doi: 10.1186/1472-6750-9-64 (PMC2720962; doi:10.1186/1472-6750-9-64)
Supplement: Additional file 5 — Sequence alignment of ITS sequences flanking the 5.8S rRNA gene from several volvocine species. [file 1472-6750-9-64-S5.pdf]

Sequence alignment of ITS sequences flanking the 5.8S rRNA gene from several volvocine species

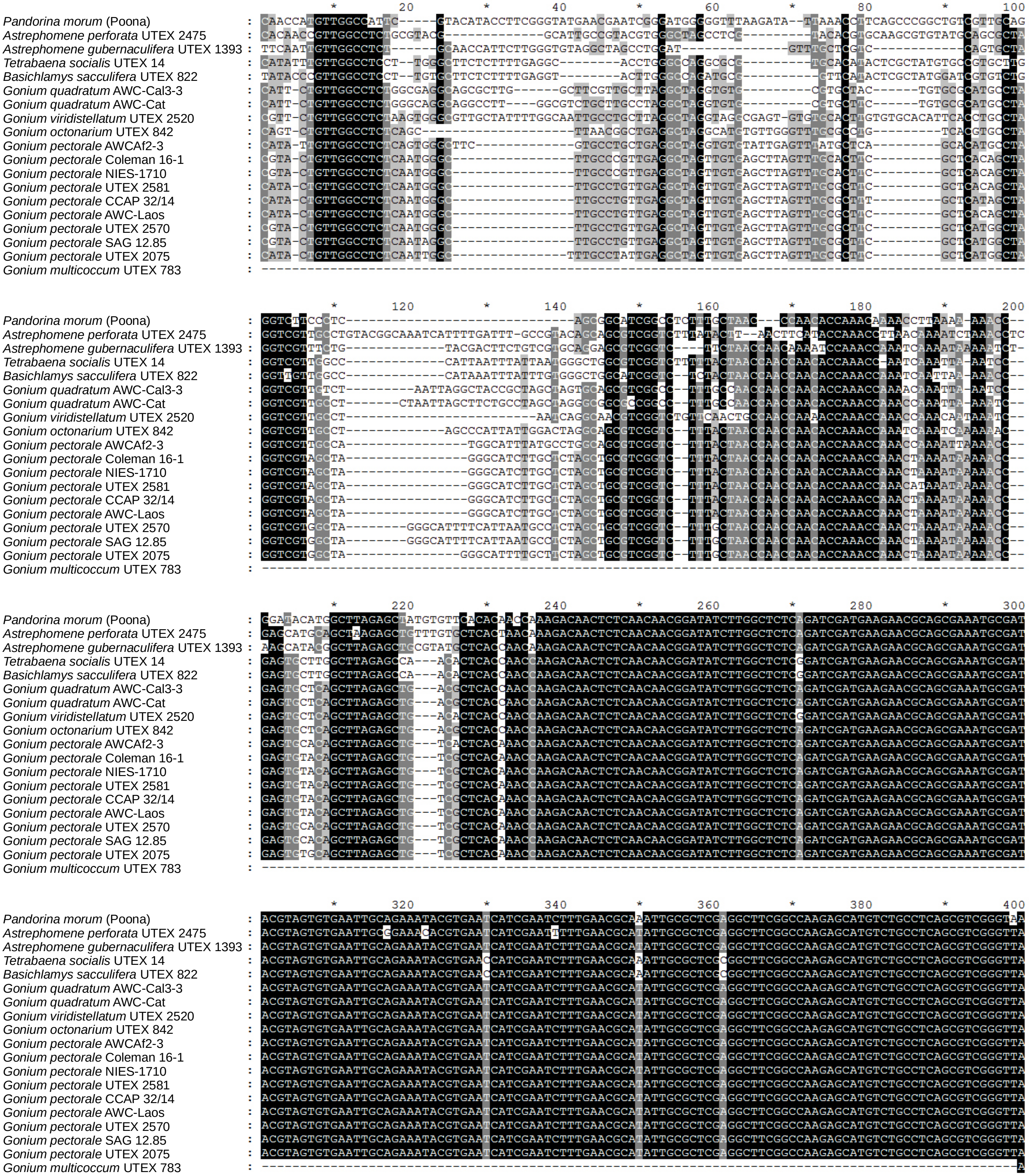

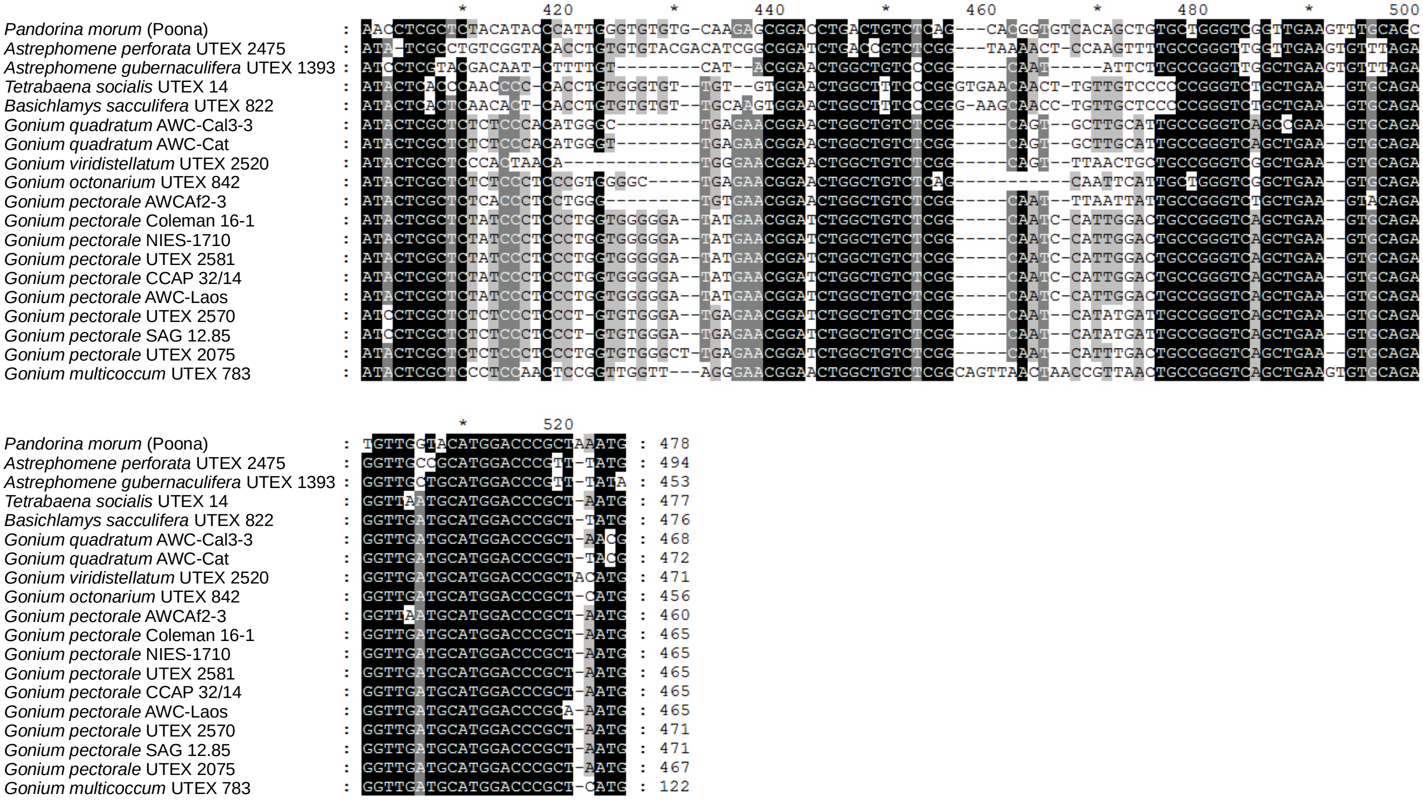

Alignment of sequences was done using the MUltiple Sequence Comparison by Log-Expectation program (MUSCLE) (Edgar, 2004). Conserved amino acid residues were shaded using GeneDoc 2.6 (Nicholas et al., 1997). White letters on black background: conserved in >90 percent of the sequences at the corresponding position; white letters on dark gray background: conserved in >70 percent of the sequences at the corresponding position; black letters on light gray background: conserved in >50 percent of the sequences at the corresponding position.

References

- Edgar RC: MUSCLE: multiple sequence alignment with high accuracy and high throughput. Nucleic Acids Res 2004, 32:1792-1797.
- Nicholas KB, Nicholas HB, Deerfield DW: GeneDoc: Analysis and visualization of genetic variation. Embnet News 1997, 4:14.
