# Supplementary material for: Stable nuclear transformation of Gonium pectorale
Source: BMC Biotechnol. 2009 Jul 10;9:64. doi: 10.1186/1472-6750-9-64 (PMC2720962; doi:10.1186/1472-6750-9-64)
Supplement: Additional file 6 — Sequence comparison of psaA, psaB and rbcL from several volvocine species. [file 1472-6750-9-64-S6.pdf]

Sequence comparison of *psa*A, *psa*B and *rbc*L from several volvocine species.

Compared species			*psa*A[a]		psaB[b]		rbcL[c]	
			% Identity	% Gaps	% Identity	% Gaps	% Identity	% Gaps
Gonium pectorale SAG 12.85	vs.	*Gonium pectorale* CCAP 32/14	### 97.83	0.00	## 94.34	1.26	### 98.47	0.00
Gonium pectorale SAG 12.85	vs.	*Gonium pectorale* NIES-1710	### 97.83	0.00	## 94.34	1.26	## 98.25	0.00
Gonium pectorale SAG 12.85	vs.	*Gonium pectorale* NIES-569	### 97.83	0.00	### 94.65	1.26	### 98.47	0.00
Gonium pectorale SAG 12.85	vs.	*Gonium quadratum* NIES-653	88.63	3.32	88.4	1.88	93.65	0.00
Gonium pectorale SAG 12.85	vs.	*Gonium viridistellatum* UTEX 2519	## 94.46	0.00	89.24	0.00	93.87	0.00
Gonium pectorale SAG 12.85	vs.	*Gonium octonarium* GO-LC-1+	92.53	0.00	86.77	5.54	# 94.09	0.00
Gonium pectorale SAG 12.85	vs.	*Gonium multicoccum* UTEX 2580	# 93.49	0.00	88.92	0.00	93	0.00
Gonium pectorale SAG 12.85	vs.	*Basichlamys sacculifera* NIES-566	90.87	0.48	88.29	0.00	91.47	0.00
Gonium pectorale SAG 12.85	vs.	*Tetrabaena socialis* NIES-571	91.11	0.48	86.83	1.88	92.12	0.00
Gonium pectorale SAG 12.85	vs.	*Astrephomene perforata* NIES-564	88.92	0.00	86.08	0.00	87.53	0.00
Gonium pectorale SAG 12.85	vs.	*Astrephomene gubernaculifera* NIES-418	89.66	0.48	86.60	3.12	88.18	0.00
Gonium pectorale SAG 12.85	vs.	*Pandorina morum* NIES-574	88.57	2.38	85.09	3.73	92.12	0.00
Gonium pectorale SAG 12.85	vs.	*Pleodorina californica* UTEX 809	90.84	0.00	89.03	1.88	91.94	0.87
Gonium pectorale SAG 12.85	vs.	*Eudorina elegans* NIES-456	91.35	0.48	# 90.82	0.00	93.25	0.87
Gonium pectorale SAG 12.85	vs.	*Eudorina unicocca* UTEX 1215	90.38	0.48	90.51	0.00	91.07	0.87
Gonium pectorale SAG 12.85	vs.	*Volvox carteri* NIES-732	89.88	0.00	88.71	1.88	91.94	0.87
Gonium pectorale SAG 12.85	vs.	*Volvox globator* UTEX 955	90.6	0.00	87.66	0.00	91.68	0.00
Gonium pectorale SAG 12.85	vs.	*Volvox aureus* NIES-541	89.64	0.00	90.51	0.00	90.85	0.87
Gonium pectorale SAG 12.85	vs.	*Chlamydomonas reinhardtii* 137C	91.11	0.48	87.66	0.00	92.14	0.44
Gonium pectorale CCAP 32/14	vs.	*Gonium pectorale* SAG 12.85	## 97.83	0.00	# 94.34	1.26	# 98.47	0.00
Gonium pectorale CCAP 32/14	vs.	*Gonium pectorale* NIES-1710	### 100.00	0.00	## 99.37	0.00	## 99.78	0.00
Gonium pectorale CCAP 32/14	vs.	*Gonium pectorale* NIES-569	### 100.00	0.00	### 99.68	0.00	### 100.00	0.00
Gonium pectorale CCAP 32/14	vs.	*Gonium quadratum* NIES-653	89.34	3.32	89.27	0.63	93.44	0.00
Gonium pectorale CCAP 32/14	vs.	*Gonium viridistellatum* UTEX 2519	# 95.42	0.00	91.17	0.63	93.65	0.00
Gonium pectorale CCAP 32/14	vs.	*Gonium octonarium* GO-LC-1+	93.01	0.00	89.59	0.63	93.87	0.00
Gonium pectorale CCAP 32/14	vs.	*Gonium multicoccum* UTEX 2580	93.01	0.00	89.87	0.00	92.78	0.00
Gonium pectorale CCAP 32/14	vs.	*Basichlamys sacculifera* NIES-566	91.59	0.48	88.96	0.63	91.68	0.00
Gonium pectorale CCAP 32/14	vs.	*Tetrabaena socialis* NIES-571	91.61	0.96	86.07	4.33	91.9	0.00
Gonium pectorale CCAP 32/14	vs.	*Astrephomene perforata* NIES-564	89.16	0.00	88.61	0.00	87.75	0.00
Gonium pectorale CCAP 32/14	vs.	*Astrephomene gubernaculifera* NIES-418	89.18	0.48	88.29	0.00	88.4	0.00
Gonium pectorale CCAP 32/14	vs.	*Pandorina morum* NIES-574	88.81	2.38	88.64	0.63	92.34	0.00
Gonium pectorale CCAP 32/14	vs.	*Pleodorina californica* UTEX 809	91.08	0.00	88.44	2.50	92.16	0.87
Gonium pectorale CCAP 32/14	vs.	*Eudorina elegans* NIES-456	91.11	0.48	89.97	1.88	93.9	0.87
Gonium pectorale CCAP 32/14	vs.	*Eudorina unicocca* UTEX 1215	90.87	0.48	89.87	0.00	91.72	0.87
Gonium pectorale CCAP 32/14	vs.	*Volvox carteri* NIES-732	90.6	0.00	88.51	3.73	92.16	0.87
Gonium pectorale CCAP 32/14	vs.	*Volvox globator* UTEX 955	91.08	0.00	89.24	0.00	91.47	0.00
Gonium pectorale CCAP 32/14	vs.	*Volvox aureus* NIES-541	89.88	0.00	89.97	1.88	91.07	0.87
Gonium pectorale CCAP 32/14	vs.	*Chlamydomonas reinhardtii* 137C	90.38	0.48	86.56	2.50	92.39	1.30

Gonium pectorale NIES-1710	vs.	*Gonium pectorale* SAG 12.85	## 97.83	0.00	# 94.34	1.26	## 98.25	0.00
Gonium pectorale NIES-1710	vs.	*Gonium pectorale* CCAP 32/14	### 100.00	0.00	## 99.37	0.00	### 99.78	0.00
Gonium pectorale NIES-1710	vs.	*Gonium pectorale* NIES-569	### 100.00	0.00	### 99.68	0.00	### 99.78	0.00
Gonium pectorale NIES-1710	vs.	*Gonium quadratum* NIES-653	89.34	3.32	89.27	0.63	93.22	0.00
Gonium pectorale NIES-1710	vs.	*Gonium viridistellatum* UTEX 2519	# 95.42	0.00	91.17	0.63	93.44	0.00
Gonium pectorale NIES-1710	vs.	*Gonium octonarium* GO-LC-1+	93.01	0.00	89.59	0.63	# 93.65	0.00
Gonium pectorale NIES-1710	vs.	*Gonium multicoccum* UTEX 2580	93.01	0.00	89.87	0.00	92.56	0.00
Gonium pectorale NIES-1710	vs.	*Basichlamys sacculifera* NIES-566	91.59	0.48	88.96	0.63	91.47	0.00
Gonium pectorale NIES-1710	vs.	*Tetrabaena socialis* NIES-571	91.61	0.96	86.07	4.33	91.68	0.00
Gonium pectorale NIES-1710	vs.	*Astrephomene perforata* NIES-564	89.16	0.00	88.61	0.00	87.53	0.00
Gonium pectorale NIES-1710	vs.	*Astrephomene gubernaculifera* NIES-418	89.18	0.48	88.29	0.00	88.62	0.00
Gonium pectorale NIES-1710	vs.	*Pandorina morum* NIES-574	88.81	2.38	88.64	0.63	92.12	0.00
Gonium pectorale NIES-1710	vs.	*Pleodorina californica* UTEX 809	91.08	0.00	88.44	2.50	91.94	0.87
Gonium pectorale NIES-1710	vs.	*Eudorina elegans* NIES-456	91.11	0.48	89.97	1.88	93.68	0.87
Gonium pectorale NIES-1710	vs.	*Eudorina unicocca* UTEX 1215	90.87	0.48	89.87	0.00	91.5	0.87
Gonium pectorale NIES-1710	vs.	*Volvox carteri* NIES-732	90.6	0.00	88.51	3.73	91.94	0.87
Gonium pectorale NIES-1710	vs.	*Volvox globator* UTEX 955	91.08	0.00	89.24	0.00	91.25	0.00
Gonium pectorale NIES-1710	vs.	*Volvox aureus* NIES-541	89.88	0.00	89.97	1.88	90.85	0.87
Gonium pectorale NIES-1710	vs.	*Chlamydomonas reinhardtii* 137C	90.38	0.48	86.56	2.50	92.17	1.30

Comparison of cDNA sequences. [a]Fragment lengths 415 bp. [b]Fragment lengths 316 bp. [c]Fragment lengths 457 bp.
###, ##, #: Highest, second highest and third highest identity value in the given data set, respectively.
