# Supplementary material for: Stable nuclear transformation of Gonium pectorale
Source: BMC Biotechnol. 2009 Jul 10;9:64. doi: 10.1186/1472-6750-9-64 (PMC2720962; doi:10.1186/1472-6750-9-64)
Supplement: Additional file 7 — Sequence comparison of ITS sequences flanking the 5.8S rRNA gene from several volvocine species [file 1472-6750-9-64-S7.pdf]

Sequence comparison of ITS sequences flanking the 5.8S rRNA gene from several volvocine species.

Compared species				ITS[a] % Identity	ITS[a] % Gaps
Gonium pectorale SAG 12.85	vs.	*Gonium pectorale* CCAP 32/14	#	94.29	2.11
Gonium pectorale SAG 12.85	vs.	*Gonium pectorale* NIES-1710		94.08	2.11
Gonium pectorale SAG 12.85	vs.	*Gonium pectorale* UTEX 2570	###	99.79	0.00
Gonium pectorale SAG 12.85	vs.	*Gonium pectorale* AWCAf2-3		88.82	3.59
Gonium pectorale SAG 12.85	vs.	*Gonium pectorale* AWC-Laos		94.08	2.11
Gonium pectorale SAG 12.85	vs.	*Gonium pectorale* Coleman 16-1		94.08	2.11
Gonium pectorale SAG 12.85	vs.	*Gonium pectorale* UTEX 2075	##	95.78	2.11
Gonium pectorale SAG 12.85	vs.	*Gonium pectorale* UTEX 2581		93.87	2.11
Gonium pectorale SAG 12.85	vs.	*Gonium quadratum* AWC-Cat		81.84	11.78
Gonium pectorale SAG 12.85	vs.	*Gonium quadratum* AWC-Cal3-3		83.4	9.92
Gonium pectorale SAG 12.85	vs.	*Gonium viridistellatum* UTEX 2520		77.41	14.93
Gonium pectorale SAG 12.85	vs.	*Gonium octonarium* UTEX 842		86.79	5.66
Gonium pectorale SAG 12.85	vs.	*Gonium multicoccum* UTEX 783		78.40	12.00
Gonium pectorale SAG 12.85	vs.	*Basichlamys sacculifera* UTEX 822		77.62	12.28
Gonium pectorale SAG 12.85	vs.	*Tetrabaena socialis* UTEX 14		77.82	12.28
Gonium pectorale SAG 12.85	vs.	*Astrephomene perforata* UTEX 2475		75.39	12.98
Gonium pectorale SAG 12.85	vs.	*Astrephomene gubernaculifera* UTEX 1393		77.69	12.37
Gonium pectorale SAG 12.85	vs.	*Pandorina morum* (Poona)		75.15	15.73
Gonium pectorale CCAP 32/14	vs.	*Gonium pectorale* SAG 12.85		94.29	2.11
Gonium pectorale CCAP 32/14	vs.	*Gonium pectorale* NIES-1710	#	98.92	0.00
Gonium pectorale CCAP 32/14	vs.	*Gonium pectorale* UTEX 2570		94.50	2.11
Gonium pectorale CCAP 32/14	vs.	*Gonium pectorale* AWCAf2-3		89.15	3.19
Gonium pectorale CCAP 32/14	vs.	*Gonium pectorale* AWC-Laos	###	99.35	0.00
Gonium pectorale CCAP 32/14	vs.	*Gonium pectorale* Coleman 16-1	#	98.92	0.00
Gonium pectorale CCAP 32/14	vs.	*Gonium pectorale* UTEX 2075		96.16	1.28
Gonium pectorale CCAP 32/14	vs.	*Gonium pectorale* UTEX 2581	##	99.14	0.00
Gonium pectorale CCAP 32/14	vs.	*Gonium quadratum* AWC-Cat		81.89	11.47
Gonium pectorale CCAP 32/14	vs.	*Gonium quadratum* AWC-Cal3-3		82.89	9.98
Gonium pectorale CCAP 32/14	vs.	*Gonium viridistellatum* UTEX 2520		77.62	14.65
Gonium pectorale CCAP 32/14	vs.	*Gonium octonarium* UTEX 842		87.05	4.46
Gonium pectorale CCAP 32/14	vs.	*Gonium multicoccum* UTEX 783		77.52	16.28
Gonium pectorale CCAP 32/14	vs.	*Basichlamys sacculifera* UTEX 822		78.02	10.08
Gonium pectorale CCAP 32/14	vs.	*Tetrabaena socialis* UTEX 14		78.51	10.84
Gonium pectorale CCAP 32/14	vs.	*Astrephomene perforata* UTEX 2475		74.09	15.93
Gonium pectorale CCAP 32/14	vs.	*Astrephomene gubernaculifera* UTEX 1393		77.64	13.21
Gonium pectorale CCAP 32/14	vs.	*Pandorina morum* (Poona)		74.61	15.82

Gonium pectorale NIES-1710	vs.	*Gonium pectorale* SAG 12.85		94.29	2.11
Gonium pectorale NIES-1710	vs.	*Gonium pectorale* CCAP 32/14	#	98.92	0.00
Gonium pectorale NIES-1710	vs.	*Gonium pectorale* UTEX 2570		94.29	2.11
Gonium pectorale NIES-1710	vs.	*Gonium pectorale* AWCAf2-3		87.21	6.08
Gonium pectorale NIES-1710	vs.	*Gonium pectorale* AWC-Laos	##	99.14	0.00
Gonium pectorale NIES-1710	vs.	*Gonium pectorale* Coleman 16-1	###	100.00	0.00
Gonium pectorale NIES-1710	vs.	*Gonium pectorale* UTEX 2075		95.52	1.28
Gonium pectorale NIES-1710	vs.	*Gonium pectorale* UTEX 2581	#	98.92	0.00
Gonium pectorale NIES-1710	vs.	*Gonium quadratum* AWC-Cat		81.12	11.85
Gonium pectorale NIES-1710	vs.	*Gonium quadratum* AWC-Cal3-3		82.48	9.98
Gonium pectorale NIES-1710	vs.	*Gonium viridistellatum* UTEX 2520		77.47	15.02
Gonium pectorale NIES-1710	vs.	*Gonium octonarium* UTEX 842		86.53	6.11
Gonium pectorale NIES-1710	vs.	*Gonium multicoccum* UTEX 783		77.52	16.28
Gonium pectorale NIES-1710	vs.	*Basichlamys sacculifera* UTEX 822		76.75	11.22
Gonium pectorale NIES-1710	vs.	*Tetrabaena socialis* UTEX 14		78.23	10.08
Gonium pectorale NIES-1710	vs.	*Astrephomene perforata* UTEX 2475		74.52	14.86
Gonium pectorale NIES-1710	vs.	*Astrephomene gubernaculifera* UTEX 1393		77.96	12.45
Gonium pectorale NIES-1710	vs.	*Pandorina morum* (Poona)		71.98	19.00

Comparison of cDNA sequences. [a]Internal transcribed spacer sequences (ITS 1 and ITS 2) flanking the 5.8S rRNA gene; fragment lengths 454 - 494 bp, except for *Gonium multicoccum* UTEX 783, which corresponds only to the 122 bp ITS 2 sequence.

###, ##, #: Highest, second highest and third highest identity value in the given data set, respectively.
