# Supplementary material for: Stable nuclear transformation of Gonium pectorale
Source: BMC Biotechnol. 2009 Jul 10;9:64. doi: 10.1186/1472-6750-9-64 (PMC2720962; doi:10.1186/1472-6750-9-64)
Supplement: Additional file 8 — Phylogeny based on psaA cDNA fragments from several volvocine species. Relationships among psaA cDNA fragments from several volvocine species. The unrooted tree was calculated using the neighbor-joining method of PHYLIP. Numbers indicate bootstrap analysis values obtained using 10000 resampled data sets. The analysis is based on the alignment given in Additional File 2. All Gonium pectorale strains are highlighted in light blue. Gonium pectorale strains used in this study are indicated by a dark blue arrow. [file 1472-6750-9-64-S8.pdf]

# Phylogeny based on *psa*A cDNA fragments from several volvocine species

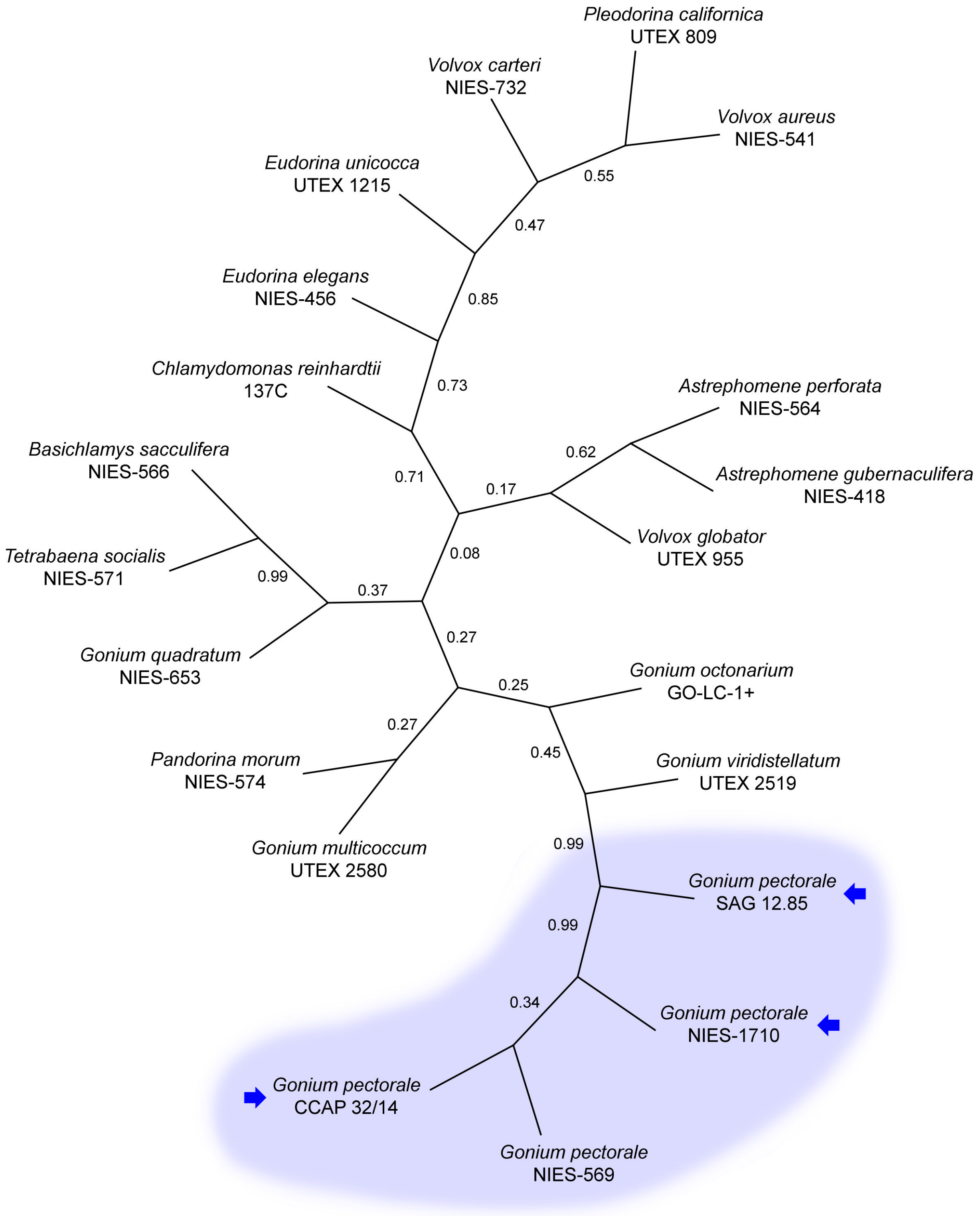


Relationships among *psa*A cDNA fragments from several volvocine species. The unrooted tree was calculated using the neighbor-joining method (Saitou and Nei, 1987) of PHYLIP (Felsenstein, 1989). Numbers indicate bootstrap analysis values obtained using 10000 resampled data sets. The analysis is based on the alignment given in Additional File 2. All *Gonium pectorale* strains are highlighted in light blue. *Gonium pectorale* strains used in this study are indicated by a dark blue arrow.


References

- Felsenstein J: Phylip - Phylogeny Inference Package (Version 3.2). Cladistics 1989, 5:164-166.
- Saitou N, Nei M: The neighbor-joining method: a new method for reconstructing phylogenetic trees. Mol Biol Evol 1987, 4:406-425.
