# Supplementary material for: Stable nuclear transformation of Gonium pectorale
Source: BMC Biotechnol. 2009 Jul 10;9:64. doi: 10.1186/1472-6750-9-64 (PMC2720962; doi:10.1186/1472-6750-9-64)
Supplement: Additional file 11 — Phylogeny based on a combined data set of psaA, psaB and rbcL cDNA fragments from several volvocine species. Relationships within a combined data set generated from the psaA, psaB and rbcL sequences from several volvocine species. The unrooted tree was calculated using the neighbor-joining method of PHYLIP. Numbers indicate bootstrap analysis values obtained using 30000 resampled data sets. The analysis is based on the alignments given in Additional Files 2, 3 and 4. All Gonium pectorale strains are highlighted in light blue. Gonium pectorale strains used in this study are indicated by a dark blue arrow. [file 1472-6750-9-64-S11.pdf]

Phylogeny based on a combined data set of *psa*A, *psa*B and *rbc*L cDNA fragments from several volvocine species

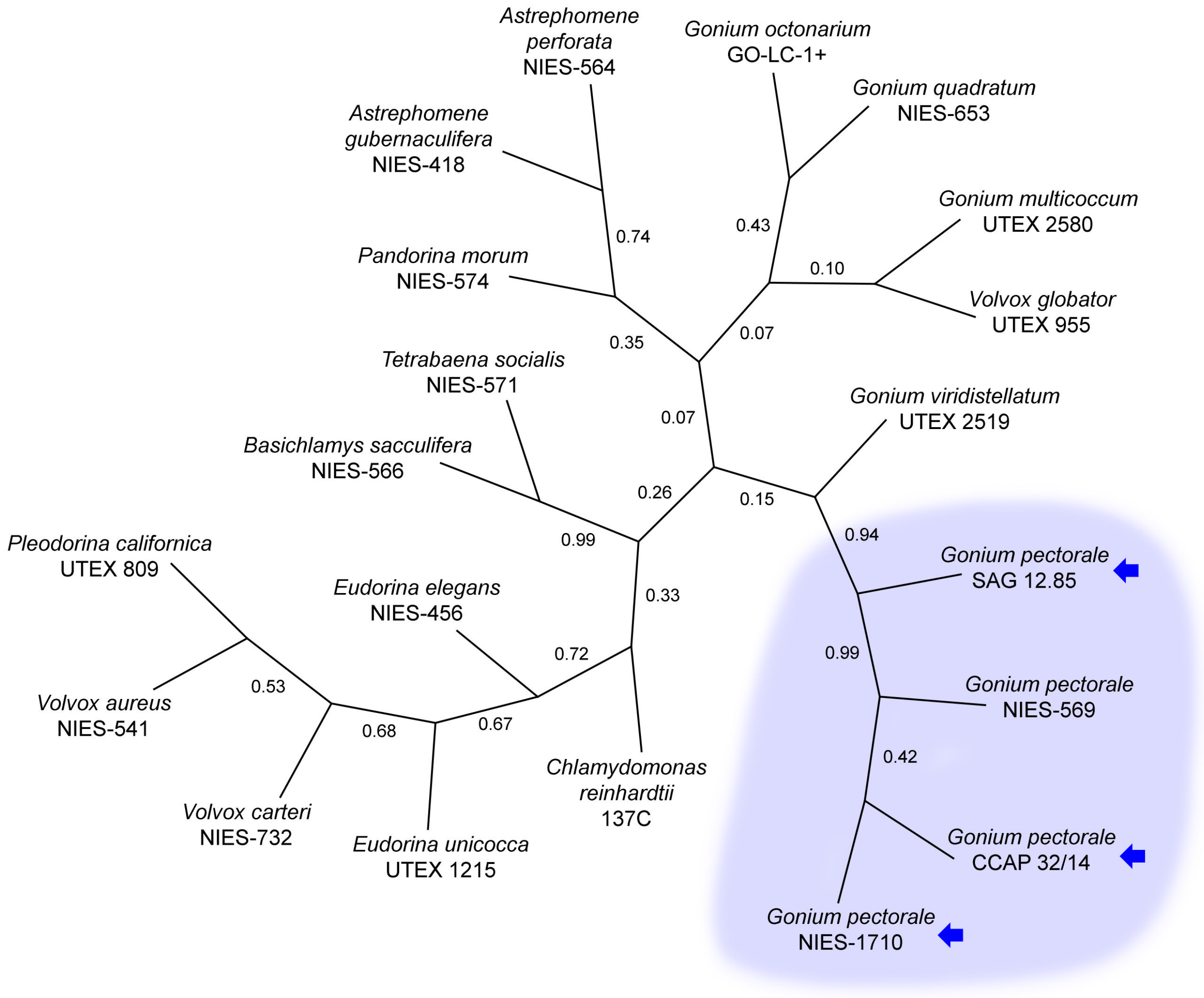

Relationships within a combined data set generated from the *psa*A, *psa*B and *rbc*L sequences from several volvocine species. The unrooted tree was calculated using the neighbor-joining method (Saitou and Nei, 1987) of PHYLIP (Felsenstein, 1989). Numbers indicate bootstrap analysis values obtained using 30000 resampled data sets. The analysis is based on the alignments given in Additional Files 2, 3 and 4. All *Gonium pectorale* strains are highlighted in light blue. *Gonium pectorale* strains used in this study are indicated by a dark blue arrow.

References

- Felsenstein J: Phylip - Phylogeny Inference Package (Version 3.2). Cladistics 1989, 5:164-166.
- Saitou N, Nei M: The neighbor-joining method: a new method for reconstructing phylogenetic trees. Mol Biol Evol 1987, 4:406-425.
